# Solvation Structure, Ion Association, and Transport in Sodium Glyme Electrolytes: From Monoglyme to Pentaglyme and Binary Mixtures

**DOI:** 10.1021/acs.jpcb.6c05191

**Published:** 2026-09-23

**Authors:** Blessing Adeleye, Valeria Bonilla, Orlando Carrillo-Bohórquez, Fedra Leonik, Ryan Jorn, Daniel Kuroda, Revati Kumar

**Affiliations:** † Department of Chemistry, 5779 Louisiana State University, 232 Choppin Hall, Baton Rouge, Louisiana 70803, United States; ‡ Department of Chemistry, 8683 Wichita State University, 206 McKinley Hall, Wichita, Kansas 67260-0051, United States

## Abstract

Glyme-based electrolytes have emerged as promising candidates for sodium-ion batteries owing to their ability to form stable solvation structures around Na^+^ via multidentate coordination with ether oxygen atoms. While the solvation and transport properties of sodium bis­(trifluoromethanesulfonyl)­imide (NaTFSI) in short-chain glymes have been systematically investigated, solutions of pentaglyme (G5) remain poorly understood and, to the best of our knowledge, are unexplored in binary glyme mixtures. Herein, molecular dynamics (MD) simulations are used to investigate the solvation structure, ion association, and transport properties of NaTFSI across six systems: monoglyme (G1), triglyme (G3), and pentaglyme (G5), as well as three binary G1:G3 mixtures of varying composition. A combination of experiments and simulations is employed to provide a comprehensive structural and dynamic characterization, including ionic conductivity, viscosity, van Hove correlation functions, and Walden analysis. The results reveal that G3 preferentially displaces G1 from the Na^+^ coordination shell, even when present in small amounts, thereby generating free G1 molecules that reduce viscosity in G1-containing systems. Analysis of the Na^+^–Na^+^ spatial correlations reveals a glyme-bridged Na^+^–G3–Na^+^ structural motif unique to G3 and persisting across all G3-containing mixtures. In contrast, G5 exhibits fundamentally different dynamical behavior from G1(0.5):G3(0.5), despite providing the same oxygen-to-Na^+^ ratio, displaying the highest viscosity and lowest conductivity among all systems studied while maintaining long-lived Na^+^-Na^+^ structural correlations. Within the binary mixtures, the G1(0.25):G3(0.75) electrolyte achieves the optimal balance, exhibiting the smallest deviation from the ideal Walden line. These findings demonstrate that molecular architecture, as well as chain length, governs electrolyte behavior, providing fundamental insights for the rational design of glyme-based sodium-ion battery electrolytes.

## Graphical abstract

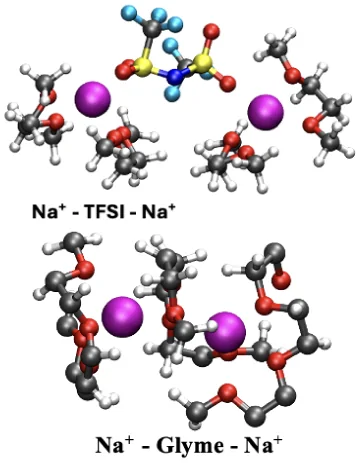

## 1 Introduction

The growing demand for reliable and sustainable energy storage technologies has intensified research into advanced rechargeable batteries for portable electronics, electric vehicles, and grid-scale applications.^1,2^ Lithium-ion batteries (LIBs) currently dominate the energy storage market because of their high energy density, lightweight design, and long cycle life.^3^ However, concerns regarding the uneven geographical distribution and rising cost of lithium resources, together with safety risks associated with flammable carbonate-based electrolytes and thermal runaway, have motivated the search for alternative battery chemistries.^4–6^

Among lithium alternatives, sodium-ion batteries (SIBs) have emerged as promising candidates for cost-effective and sustainable large-scale energy storage applications. Sodium is approximately 1000 times more abundant than lithium, inexpensive to source, and evenly distributed across the globe.^7,8^ While sodium has a larger ionic radius and higher atomic mass compared to lithium, its electrochemical potential (−2.71 V vs SHE, standard hydrogen electrode)^9^ is only slightly lower than that of lithium (−3.04 V vs SHE),^10^ making it suitable for many stationary and medium-scale applications where constraints on size and weight are less stringent. The similarity between sodium and lithium enables SIBs to operate through comparable “rocking-chair” mechanisms and to share similar electrode materials with LIBs.^11,12^ These advantages, combined with cost-effectiveness and enhanced safety, position SIBs as an attractive candidate for sustainable energy storage, though achieving their full potential depends critically on the development of suitable electrolyte systems.

The electrolyte plays a central role in determining the charging rate and storage capacity fade in rechargeable batteries across repeated charge cycles.^13^ An ideal electrolyte for SIBs should be chemically, electrochemically, and thermally stable, provide high ionic conductivity and electronic insulation, and have low toxicity.^4^ Carbonate-based electrolytes, which are widely used in LIBs with solvents such as ethylene carbonate (EC) and propylene carbonate (PC),^14,15^ are natural candidates owing to their high dielectric constants and ability to form passivating interphases on electrode surfaces.^16^ However, they suffer from several drawbacks in SIBs, such as continuous formation and dissolution of the solid electrolyte interphase (SEI), and safety concerns from flammability.^17^ These challenges have motivated the search for alternative electrolyte systems that can provide both stability and efficient ion transport.

Ether-based electrolytes have emerged as important candidates for sodium-ion batteries due to their ability to coordinate alkali metal cations through ether oxygen atoms, which provide stable solvation environments and enable ion transport.^6,18^ These systems exist in both liquid and solid forms. In the solid state, poly­(ethylene oxide) (PEO) has been extensively investigated as a solid polymer electrolyte (SPE).^19^ The repeating −CH_2_–CH_2_–O– units of PEO solvate sodium ions, and conduction occurs through cation hopping between ether oxygens along the polymer backbone.^20^ PEO-based SPEs offer advantages such as good mechanical stability and suppression of metal dendrite growth, making them attractive for safer battery operation.^21,22^ However, their low ionic conductivity at room temperature,^23^ caused by polymer crystallinity that restricts chain mobility, remains a major limitation.^24,25^ Hence, PEO-based SPEs often require elevated operating temperatures to access the amorphous region, where ion transport is more efficient. For example, Qi et al. proposed an SPE composed of PEO and sodium bis­(fluorosulfonyl)­imide [Na­[N­(SO_2_F)_2_]], referred to subsequently as NaFSI, with a relatively high conductivity of 4.1 × 10^–4^ S cm^–1^, but at an elevated temperature of 80 °C. Interestingly, they also observed that the salt, apart from serving as a sodium source, promoted the interfacial stability between the electrolyte membrane and the metallic sodium.^26^

Success with PEO-based SPEs has motivated the exploration of liquid analogues that share the same ether-based chemistry, but avoid the crystallinity problem. The family of glyme ethers, also known as glycol dimethoxy ethers, form shorter chain versions of PEO which are liquid at room temperature and consist of repeating ethylene glycol units with methyl groups at both ends.^27,28^ The glyme family possesses the general formula CH_3_(OCH_2_CH_2_)_n_OCH_3_ and the most studied variants include G1 (monoglyme, *n* = 1), G2 (diglyme, *n* = 2), G3 (triglyme, *n* = 3), G4 (tetraglyme, *n* = 4), and G5 (pentaglyme, *n* = 5). Glyme ethers maintain the strong cation coordination characteristic of PEO while remaining fluid at room temperature. Their chelating ability provides stable solvation environments and enables sodium cointercalation into graphite,^29^ which is otherwise inactive with carbonate electrolytes.^30,31^ Glymes form well-defined complexes with sodium salts, such as NaFSI, NaTFSI, NaOTf, and NaPF6,^32–35^ have high electrochemical stability^36,37^ and relatively wide potential windows. Glymes also provide an ideal platform for probing solvation structures and transport dynamics in sodium electrolytes, both experimentally and computationally.^38^

Wahlers et al. developed a force field for NaOTf in G2, derived from *ab initio* molecular dynamics using a variational force-matching approach. This model successfully reproduced solvation structures across concentrations and was shown to be transferable to other glymes.^39^ Li et al. tested the model’s transferability to NaTFSI in glymes from G1 to G4 and confirmed that the force field accurately captured sodium-anion coordination. Their study also revealed systematic trends with ether chain length: shorter glymes (G1, G2) exhibited stronger ion association and aggregation, while longer glymes (G3, G4) promoted more stable, solvent-dominated coordination environments, and lower conductivity.^40^ Furthermore, they showed that solvation properties did not change monotonically with chain length. For example, sodium ions with just one anion in the solvation shell are seen for G1, G2, and G4 but not G3. These studies demonstrate the dual importance of anion identity and glyme chain length in shaping both solvation structure and transport dynamics.

Although the solvation and transport behavior of sodium salts in G1 through G4 have been systematically investigated, G5 remains largely unexplored. With six ether oxygen atoms, G5 provides an opportunity to examine whether increasing glyme chain length starts to bridge the gap between short molecular glymes and polymeric systems, such as PEO, while maintaining liquid-state transport properties. Furthermore, a 1:1 binary mixture of G1 and G3 provides the same total number of ether oxygen atoms as G5, but with a fundamentally different molecular architecture. In G5, all six ether oxygens are connected along a single extended chain, whereas in the mixture they are distributed in two separate molecules. This comparison provides an opportunity to examine how molecular arrangement influences solvation structure and ion transport despite an equivalent number of coordination sites. In this paper, we systematically investigate the solvation structure, ion association, and transport properties of NaTFSI in G5 and binary G1:G3 mixtures of varying composition using molecular dynamics simulations to elucidate how glyme chain length and molecular architecture influence sodium-ion coordination and transport. Furthermore, comparison of simulations and measurements of the conductivity and viscosity serve as a critical test of the transferability of the force field for both longer glymes and mixed-solvent systems. The paper is divided into the following sections: Section 2 outlines the methodology used while the results are presented and discussed in Section 3 and the conclusions are summarized in Section 4.

## 2 Methodology

### 2.1 Experimental Methods

#### 2.1.1 Synthesis of G5

To a suspension of sodium metal (1.8 g, 78 mmol) in dry THF (10 mL), 2-methoxyethanol (5.6 mL, 5.4 g, 71 mmol) was added dropwise at 0 °C under stirring. The mixture was stirred at this temperature for 30 min. Subsequently, a solution of tri­(ethylene glycol) ditosylate (6.5 g, 14 mmol) in THF (20 mL) was added slowly at 0 °C. The reaction mixture was stirred at room temperature for 24 h. After completion, the reaction was cooled, carefully quenched with water (30 mL), and THF was removed under reduced pressure. The crude product was extracted with DCM (3 × 30 mL). The combined organic layers were dried over MgSO_4_, filtered, and concentrated in vacuo. The residue was purified by silica gel column chromatography using ethyl acetate/hexanes (9:1), ethyl acetate, and ethyl acetate/methanol (9:1) as eluents. The product was obtained as a light yellow oil (2.3 g, 8.6 mmol, 61% yield). ^1^H NMR (400 MHz, CDCl_3_): δ 3.66–3.63 (m, 16H), 3.56–3.55 (m, 4H), 3.38 (s, 6H).^41^

#### 2.1.2 Sample Preparation

All samples were prepared in a nitrogen-filled glovebox to minimize water contamination. G1 (+99%, ThermoScientific) and G3 (99%, ThermoScientific) were dried in 4 Å molecular sieves for at least 48 h before use. G5 was synthesized using the method described above, and later dried in 4 Å molecular sieves over 72 h. The water content of the three solvents was below 50 ppm, as determined by Karl Fischer titration. NaTFSI (98%, AmBEED) was dried in a vacuum oven at 120 °C for 24 h.

#### 2.1.3 Viscosity and Ionic Conductivity Measurements

The viscosity measurements of the solutions were performed at 20 °C using the Brookfield DVI-II+Pro. The accuracy of the viscometer was estimated by measuring ultrapure water and G3 at 20 °C, with less than 5% error when compared to reported values.

Ionic conductivity measurements were performed using the potentiostatic electrochemical impedance spectroscopy (EIS-POT) technique in a nitrogen-filled glovebox. The WaveDriver 100 Potentiostat (Pine Research) was used with a ceramic platinum screen printed electrode (Pt-SPE, RRPE2011PT-6 PineResearch), consisting of 2 mm platinum working electrode (WE), a platinum counter electrode (CE), and a Ag/AgCl pseudoreference electrode. The electrochemical cell was allowed to equilibrate for at least 15 min to stabilize the potential of the pseudo-reference electrode. The electrochemical cell constant for the ionic conductivity vs EIS-measured cell resistance was determined using potassium chloride (KCl) solutions.

### 2.2 Computational Methods

#### 2.2.1 Classical Molecular Dynamics Simulations

Classical molecular dynamics (MD) simulations of six different systems were performed using the LAMMPS software package.^42^ The molecular structures of G1, G3, G5, and NaTFSI (Figure 1) were constructed using the Avogadro software.^39^ Initial cubic simulation boxes, with a side length of approximately 40 Å, were generated using Packmol,^40^ where molecules were placed using a random packing algorithm.^43^ The systems were comprised of NaTFSI dissolved in pure G1, G3 and G5, as well as in three binary G1:G3 mixtures with molar compositions of G1(0.75):G3(0.25), G1(0.5):G3(0.5), and G1(0.25):G3(0.75).

**Figure 1:**
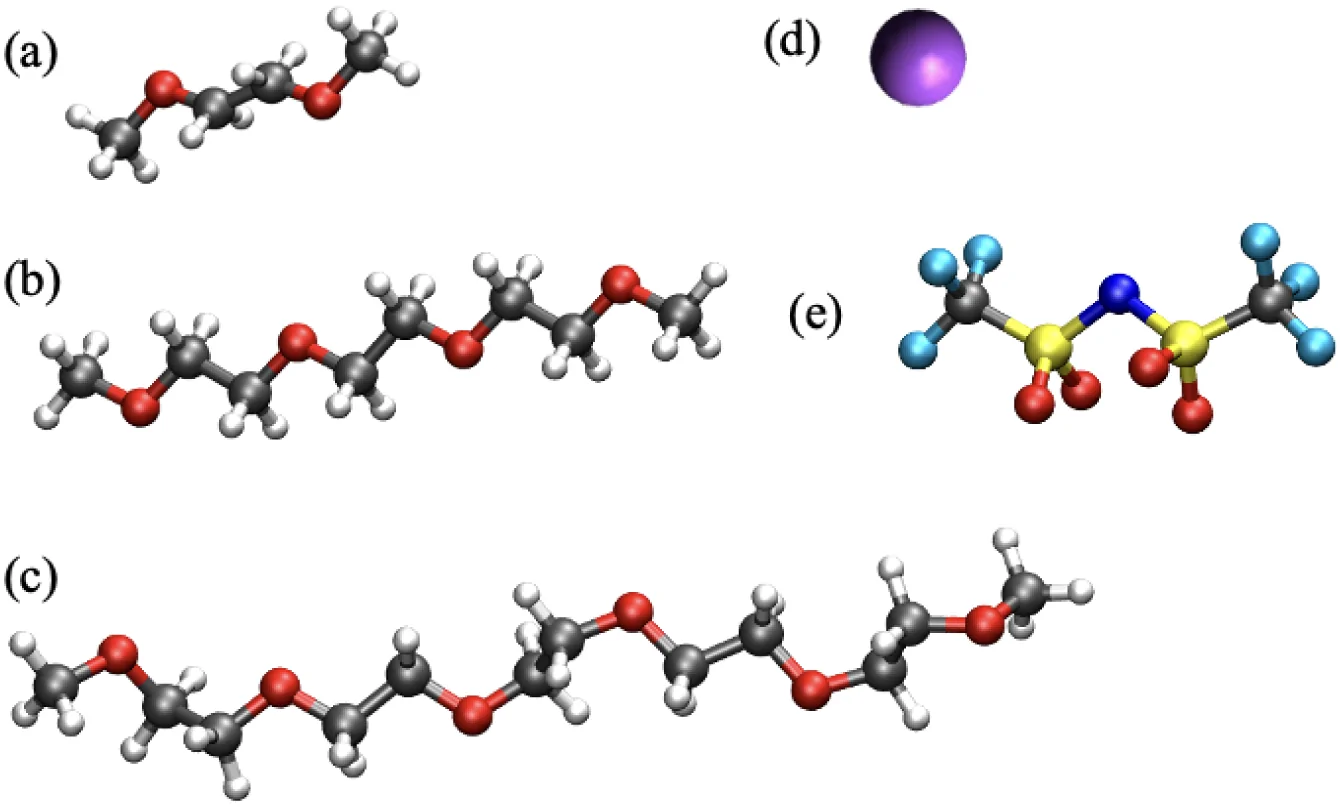
Molecular structure of (a) G1, (b) G3, (c) G5, (d) sodium cation, and (e) TFSI anion. Atoms are colored as follows: Na (purple), C (gray), O (red), H (white), S (yellow), F (cyan), and N (blue).

The number of NaTFSI formula units was fixed at 58 across all systems and the ratio of Na^+^ to glyme ether oxygen atoms was maintained at 1:12, resulting in a NaTFSI concentration of around 1.5 M across all systems. The number of glyme molecules in each system varies accordingly and is summarized in Table S1 of the (SI).

The intermolecular interactions were described using the Polymer Consistent Force Field (PCFF)^44,45^ for the glyme molecules. Ion–solvent and ion–ion interactions were treated using a modified model from a previous study.^39^ Partial charges for the glymes and TFSI^–^ were adopted from previous work.^39,46^ For G1, the partial charges were scaled by a factor of 1.1 relative to the original PCFF values to account for polarization and charge transfer effects^47^ and to bring the simulated Na^+^–O­(glyme) radial distribution functions into agreement with *ab initio* molecular dynamics reference data, as validated in Figure S1 of the SI. This correction was applied only to G1, while the charges for G3 and G5 were retained as defined in the PCFF model. A real-space cutoff of 6.0 Å was applied to both Lennard-Jones and Coulombic interactions, and long-range electrostatic interactions were treated using the particle–particle particle-mesh (PPPM) Ewald summation method.^48^

Each system was first equilibrated in the NPT ensemble at 300 K and 1 atm using a Nosé–Hoover thermostat and barostat with relaxation times of 0.1 and 1 ps, respectively, and a time step of 1 fs. To facilitate proper structural relaxation and avoid kinetic trapping during equilibration, the charges on Na^+^ and TFSI^–^ were gradually scaled by factors of 0.6, 0.8, and 1.0 over successive 2 ns intervals, followed by an additional 30 ns equilibration at full ionic charges, resulting in a total equilibration time of 36 ns. Production runs of 200 ns were subsequently performed in the NVE ensemble, initiated from NPT-equilibrated configurations, with a time step of 1 fs. The use of the NVE ensemble for production ensures that ionic dynamics are not perturbed by a thermostat, which is critical for the accurate calculation of transport properties.

#### 2.2.2 Replica Exchange Molecular Dynamics

To improve the sampling of equilibrium solvation structures and validate the solvation shell fractions obtained from the classical MD trajectories, replica exchange MD (REMD) simulations were performed for each system.^49^ The REMD trajectories also served as additional starting configurations for the nonequilibrium molecular dynamics (NEMD) conductivity simulations. In REMD, multiple independent replicas of the system are simulated simultaneously at different temperatures. At regular intervals, swap attempts are made between replicas at neighboring temperatures according to the Metropolis criterion. This allows configurations to escape local energy minima by temporarily accessing higher-temperature ensembles, resulting in more thorough sampling of the equilibrium phase space at the target temperature.^50^ For each system, 10 replicas were distributed uniformly across a temperature range of 300 to 370 K using initial structures from the previous classical MD and simulated in the NVT ensemble for 50 ns each, with swap attempts made every 1000 time steps. To verify adequate replica exchange and sampling, the temperature evolution as a function of simulation time was monitored for three representative replicas initialized at the lowest (300 K), intermediate (330 K), and highest (370 K) temperatures. The temperature evolution profiles for representative replicas of the glyme mixture systems are shown in Figure S2.

#### 2.2.3 Ab-Initio Molecular Dynamics

To validate the modified partial charges assigned to G1 in this work, AIMD simulations were performed.^51^ First, classical MD simulations of G1 were used to generate initial configurations for subsequent AIMD calculations. A small simulation box containing 18 G1 molecules and 3 NaTFSI ion pairs, with an edge length of approximately 16 Å, was constructed using the methodology described above. The system was equilibrated in the NVT ensemble for 4 ns, followed by 10 ns in the NPT ensemble, and then propagated for 10 ns in the NVE ensemble for production. Four independent configurations were extracted from the resulting trajectory and used as initial structures for AIMD simulations.

The CP2K software package^52^ was used to run the AIMD simulations with a time step of 0.5 fs. The Perdew–Burke–Ernzerhof (PBE) exchange-correlation functional,^53^ together with the Grimme D3 dispersion correction,^54^ was used in combination with the TZV2P basis set and Goedecker–Teter–Hutter (GTH) pseudopotentials^55^ for the core electrons. The plane-wave cutoff was set to 600 Ry with a relative cutoff of 60 Ry. The Nosé–Hoover thermostat and barostat, with time constants of 50 and 500 fs, respectively, were used for NPT equilibration. Starting from the MD-derived configurations, four independent AIMD simulations were performed to improve sampling, each equilibrated for 10 ps in the NPT ensemble at 300 K and 1 atm, followed by 10 ps of NVE dynamics. Radial distribution functions (RDFs) for the main interactions were extracted from these trajectories and compared with those obtained from classical MD.

### 2.3 Structural and Dynamical Analyses

To understand how glyme chain length governs the solvation environment and transport behavior of NaTFSI-based electrolytes, the structural and dynamic properties were examined. The structural properties provide a quantitative description of how Na^+^ interact with glyme molecules and TFSI^–^ anions at the molecular level. The dynamical properties reveal how these local structural environments translate into macroscopic transport behavior and were characterized using the analyses described below.

#### 2.3.1 Radial Distribution Functions and Coordination Numbers

Radial distribution functions (RDFs) were computed from the classical MD production trajectories to characterize the local solvation environment and ion interactions in each glyme system. The RDFs were also computed from REMD simulations and compared to the canonical MD simulations to demonstrate convergence of the 200 ns canonical MD simulations. The RDF describes the probability of finding a particle at distance *r* from a reference particle relative to that expected for a uniform distribution at the same density.^56^ RDFs were computed between Na^+^ and three key atomic species: the glyme oxygen atoms [Na–O­(glyme)], the oxygen atoms of TFSI^–^ [Na–O­(TFSI)], and the sulfur atoms of TFSI^–^ (Na–S), providing direct insight into how Na^+^ coordinates with both the solvent and the anion molecules. RDFs were calculated over the entire production run using the radial pair distribution tool in VMD.^57^ To further quantify the extent of solvation, coordination number *n­(r)* was determined by integrating the RDFs up to the first minimum, which defines the outer boundary of the first solvation shell:^17^

1 $$n\left(r\right)=\int_{0}^{r_{\min}}4\pi r^{2}\rho g\left(r\right)dr$$

where *ρ* is the number density of the coordinating species and *g­(r)* is the associated radial distribution function.

#### 2.3.2 Diffusion Coefficients

The self-diffusion coefficients of Na^+^ and TFSI^–^ were determined separately from the production trajectories using the mean squared displacement (MSD) method, which relates particle displacement over time to long-time diffusive motion. The MSD of a species at time *t* is defined as

$$\langle \Delta r(t{)}^{2}\rangle =\frac{1}{N}\overset{N}{\underset{i=1}{\sum }}{|r_{i}\left(t\right)-r_{i}\left(0\right)|}^{2}$$ 2

where *N* is the number of particles of the species considered and **r**_i_ *(t)* is the position vector of particle *i* at time *t*. According to the Einstein relation,^58^ the self-diffusion coefficient *D* is obtained from the slope of the MSD in the long-time diffusive regime.

#### 2.3.3 Ionic Conductivity

The Nernst–Einstein ionic conductivity, *σ_NE_*, also referred to as the uncorrelated conductivity, was calculated using the Nernst–Einstein relation.^59^ This approach assumes that the motions of cations and anions are independent and uncorrelated, allowing conductivity to be expressed directly in terms of their self-diffusion coefficients:

3 $$\sigma_{NE}=\frac{e^{2}}{Vk_{B}T}\left(N_{+}q_{+}^{2}D_{+}+N_{-}q_{-}^{2}D_{-}\right)$$

where *e* is the elementary charge, *V* is the simulation box volume, *k*_*B*_ is the Boltzmann constant, and *T* is the temperature. *D_+_* and *D*_*–*_ represent the self-diffusion coefficients of Na^+^ and TFSI^–^ respectively, *q*_*+*_ and *q_–_* are their respective charges, and *N*_+_ and *N–* are the total number of each ion species.^17^ The correlated ionic conductivity, λ, was evaluated from equilibrium molecular dynamics trajectories using the charge–charge displacement correlation function^60,61^ which explicitly accounts for cross-correlations between the motions of distinct ions and is defined as

$$\lambda =\underset{t⃗\infty }{\lim}\frac{e^{2}}{6Vk_{B}Tt}\overset{N}{\underset{\alpha ,\beta }{\sum }}Z_{\alpha }Z_{\beta }\left[r_{\alpha }\left(t\right)-r_{\alpha }\left(0\right)\right]\cdot \left[r_{\beta }\left(t\right)-r_{\beta }\left(0\right)\right]$$ 4

where *e* is the elementary charge, *V* is the simulation cell volume, *k*_*B*_ is the Boltzmann constant, *T* is the temperature, *Z*_α_ and *Z*_β_ are the ionic charges, and **r**_α_ *(t)* is the displacement vector of ion α at time *t*. The total conductivity can be decomposed into contributions from like-charge and cross-charge correlations:

5 $$\lambda =\lambda_{++}+\lambda_{--}+\lambda_{+-}$$

where λ_++_ and λ_––_ correspond to cation–cation and anion–anion correlations, respectively, and are always positive. The cross-term λ_+–_ captures the correlation between cation and anion motions and can be positive or negative depending on whether Na^+^ and TFSI^–^ move in opposing or correlated directions. When Na^+^ and TFSI^–^ move together as ion pairs, λ_+–_ is negative and reduces the net conductivity below the Nernst–Einstein estimate. When their motions are anticorrelated, λ_+–_ is positive and contributes favorably to charge transport. The cross-term λ_+–_ is therefore the key quantity connecting ion association to transport efficiency and is the primary focus of the conductivity analysis presented in this work.

To provide confirmation of the correlated ionic conductivity, nonequilibrium molecular dynamics (NEMD) simulations were performed for each system at a constant temperature of 300 K. The nonself cation–cation and anion–anion correlations often take significantly longer time to converge and hence NEMD offers an efficient alternative to estimating the correlated conductivities. Starting configurations were drawn from both the REMD simulations and the NVE production runs. An external electric field of 0.05 V/Å was applied along the *z*-direction for 100 ns, and the resulting ionic current was used to determine the conductivity of each electrolyte. The applied field strength was selected to be within the linear response regime, as shown in Figure S2 ensuring that the computed conductivity is field-independent and directly comparable to equilibrium estimates.^62,63^ Statistical uncertainties for the calculated diffusion coefficients and NEMD conductivities were estimated using block averaging and reported as the standard deviation of the mean. Diffusion coefficients were computed using 4 blocks, each 50 ns, and NEMD conductivity using 4 blocks of 25 ns each.

#### 2.3.4 Viscosity

The shear viscosity of each system was computed using the Green–Kubo formalism,^64–67^ in which the viscosity is obtained from the time integral of the autocorrelation function of the off-diagonal stress tensor components:

6 $$\eta =\frac{V}{k_{B}T}\int_{0}^{\infty }\langle P_{\alpha \beta }\left(t\right)\cdot P_{\alpha \beta }\left(0\right)\rangle dt$$

where *V* is the simulation volume, *k*_*B*_ is the Boltzmann constant, *T* is the temperature, and *P_αβ_* represents the off-diagonal elements of the stress tensor. Simulations for each system were carried out for 200 ns of production, with the components of the stress tensor recorded every 10 fs for Green–Kubo viscosity calculations. To improve statistical reliability, the trajectories were divided into 80 independent blocks, and the running integrals of the stress autocorrelation function were averaged across blocks. The block-averaged viscosity values were used to estimate the standard deviation, providing a measure of uncertainty for each reported viscosity. The averaged running integrals were further fitted to a double-exponential function,^66,68^ weighted by the power-law behavior of the standard deviation, to extract converged long-time viscosity values. This protocol was adopted to ensure robust and converged viscosity estimates, given the slow decay of stress autocorrelation functions characteristic of viscous glyme-based electrolytes.^69^

#### 2.3.5 Van Hove Correlation Function

To probe the time evolution of Na^+^–Na^+^ spatial correlations and gain insight into the dynamic rearrangement of the ionic network, the distinct part of the van Hove correlation function, *G_d_(r, t)*, was computed from the production trajectories for each system using the TRAVIS software package.^70,71^ The distinct van Hove function is defined as

$$G_{d}\left(r,t\right)=\frac{1}{N}\overset{N}{\underset{i=1}{\sum }}\overset{N}{\underset{j\neq i}{\sum }}\langle \delta \left(r-\left|r_{i}\left(t\right)-r_{j}\left(0\right)\right|)\right\rangle$$ 7

where *N* is the number of Na^+^ ions, **r** *_i_(t)* is the vector position of ion *i* at time *t*, and **r**_*j*_(0) is the position of ion *j* at the reference time.^72^ At *t* = 0, *G_d_(r, t)* reduces to the Na^+^-Na^+^ radial distribution function, providing a direct link between the static structure and its time evolution. By evaluating *G_d_(r, t)* at successive time intervals, the rate at which Na^+^–Na^+^ structural correlations decay can be assessed, offering insight into the persistence of ion clustering and its relationship to transport behavior across the glyme series.

## 3 Results and Discussion

### 3.1 Solvation Structure

The force field used in this work was derived from a previously developed model parametrized against AIMD simulations of 1.5 M NaOTF in G2 and subsequently extended to longer glymes. Its applicability to TFSI^–^ based electrolytes was later assessed through comparison with AIMD simulations of 1.5 M NaTFSI in G2. For the present work, the model was reassessed for monoglyme using AIMD simulations of G1. Comparisons of the Na–O­(glyme), Na–N­(TFSI), and Na–S­(TFSI) radial distribution functions revealed differences between the AIMD results and those obtained using the original force field parameters, with the original charges overestimating the Na^+^–O­(glyme) coordination relative to the AIMD reference data. Scaling the G1 partial charges by a factor of 1.1 yielded significantly better agreement with the AIMD reference data, as shown in Figure S1 of the SI, and were adopted in subsequent simulations.

The local solvation environment of Na^+^ was first characterized through radial distribution functions for the Na–S, Na–O­(TFSI), and Na–O­(glyme) atom pairs across all six systems (Figure 2). The RDFs obtained from the REMD simulations are very similar and are plotted in Figure S2 in the SI. Across the entire series, the Na–O­(glyme) and Na–O­(TFSI) RDFs exhibit a sharp first peak at approximately 2.3 Å, while the Na–S RDF shows a first peak at approximately 3.6 Å, consistent with TFSI^–^ coordinating Na^+^ through its oxygen atoms. The near-identical peak positions across all systems confirm that glyme chain length does not alter the fundamental Na^+^ coordination geometry. The substantially higher intensity of the Na–O­(glyme) first peak relative to Na–O­(TFSI) across all systems establishes the strong chelation of the Na^+^ by the glymes, with the total oxygen coordination number falling consistently in the range of 6–7 across all systems, in agreement with previous simulation studies of NaTFSI in glyme electrolytes.^17^ Despite this universally similar total coordination, the molecular composition of the first solvation shell varies dramatically across systems, as revealed by the solvation shell fraction analysis presented in Figure 3.

**Figure 2:**
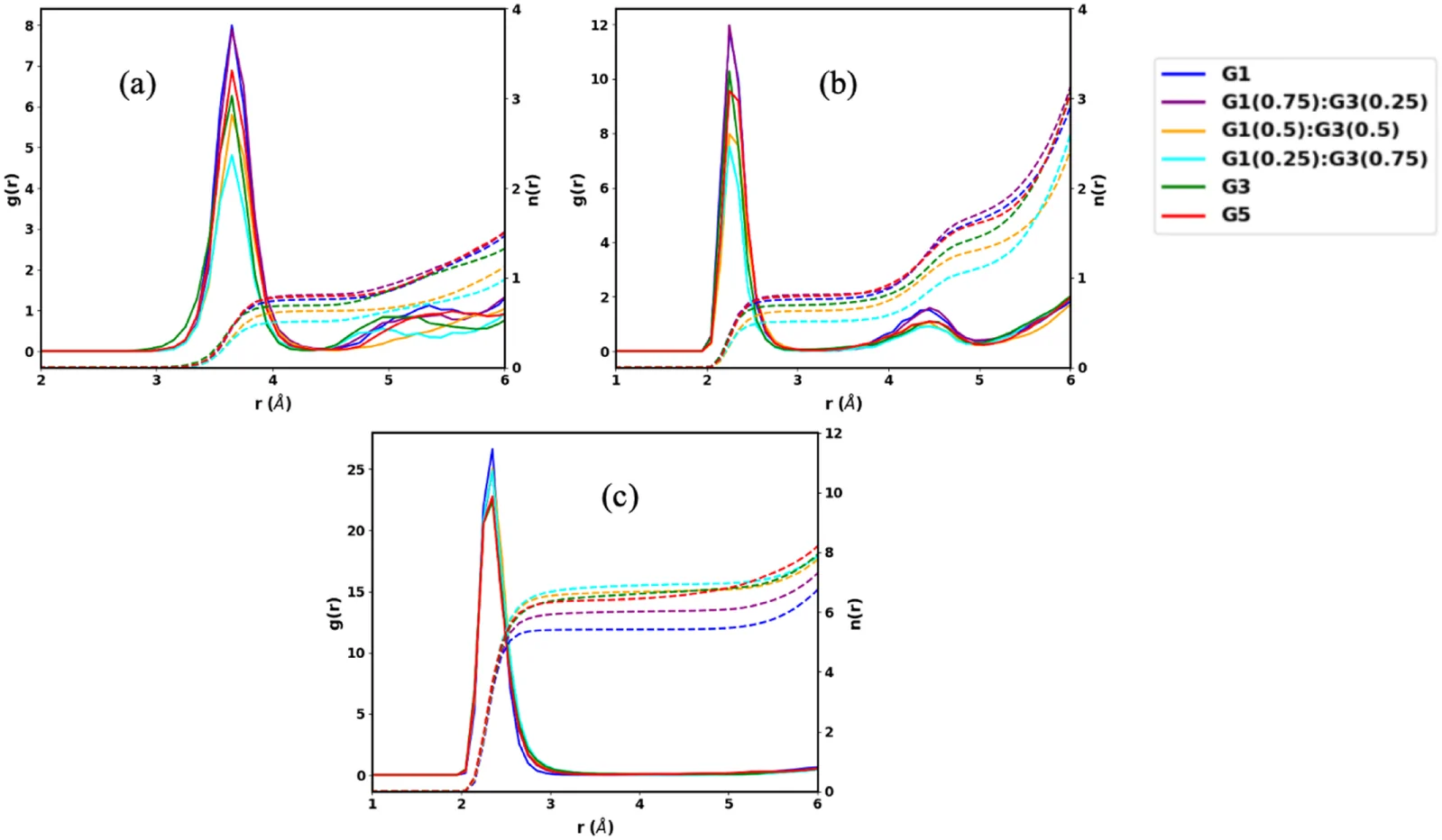
Radial distribution functions, *g­(r)* (solid lines), and coordination numbers, *n­(r)* (dashed lines), between Na^+^ and (a) sulfur atoms of TFSI^–^, (b) oxygen atoms of TFSI^–^, and (c), glyme oxygen atoms, across G1, G3, G5, and mixed-solvent systems.

**Figure 3:**
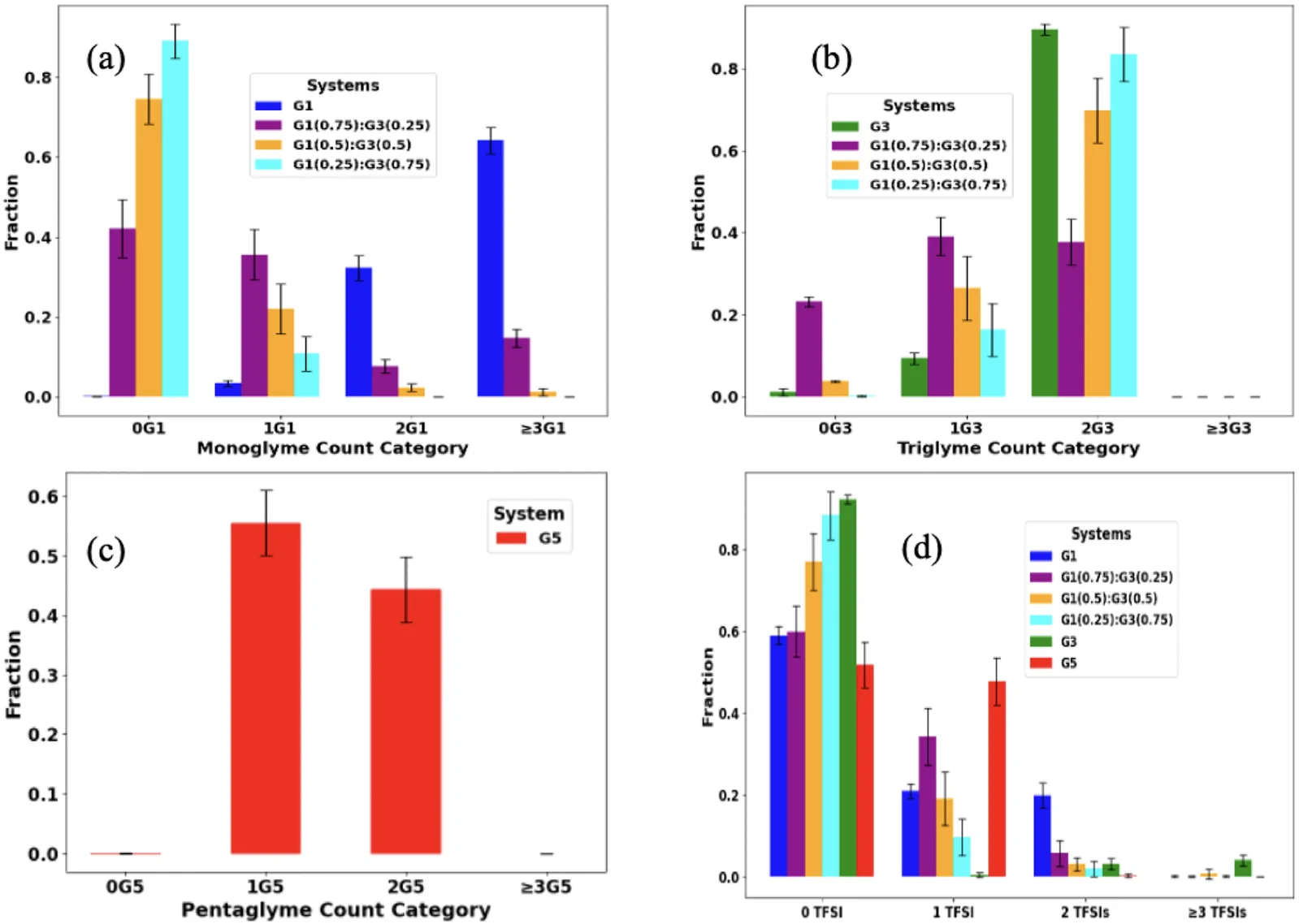
Distribution of the fraction of (a) Na^+^ with different number of G1 in their first solvation shell in pure G1 electrolytes and the G1–G3 mixtures, (b) Na^+^ with different number of G3 in the first solvation shell in the pure G3 and G1–G3 mixture electrolytes, (c) Na^+^ with different number of G5 in the G5 electrolyte, and (d) Na^+^ with different number of TFSI species for each of the electrolytes.

In monoglyme (G1), the Na^+^ solvation shell is assembled from multiple short-chain molecules, with 64% of Na^+^ coordinated by three or more G1 molecules (≥3G1) and 32% by two, reflecting the need for multiple molecules to collectively satisfy the 6–7 oxygen Na^+^ coordination requirement (Figure 3). The detailed solvation shell composition shown in Table S2 reveals that 41% of Na^+^ have at least one TFSI^–^ in their first coordination sphere, confirming significant ion association in G1. The two most populated environments are ≥ 3G1 + 0TFSI (50%) and 2G1 + 2TFSI (19%), indicating that when TFSI^–^ does coordinate Na^+^, it tends to do so alongside another TFSI^–^ rather than alone (Figure 3, Table S2). Moving to the mixtures with triglyme, introducing even a small amount of G3 disrupts the multi-G1 coordination environment disproportionately. In the G1(0.75):G3(0.25) mixture, despite G1 constituting 75% of the solvent by mole fraction, 42% of Na^+^ already have no G1 in their first solvation shell. As the amount of G3 increases by mole fraction, the trend toward displacing G1 from the Na^+^ solvation shell is clear with 75% of structures in G1(0.5):G3(0.5) lacking a G1 oxygen and 89% in G1(0.25):G3(0.75). The most populated solvation environment in these mixtures is 0G1 + 2G3 + 0 TFSI, accounting for 38%, 70%, and 84% of Na^+^ in G1(0.75):G3(0.25), G1(0.5):G3(0.5), and G1(0.25):G3(0.75) respectively (Table S2). The preferential solvation by G3 reflects the thermodynamic advantage of multidentate chelation, where a single G3 molecule donating four ether oxygens is entropically favored over multiple G1 molecules competing for the same coordination sites. As a direct consequence, the G1 molecules displaced from the Na^+^ solvation shell populate the bulk as free, uncoordinated species. Molecular snapshots of the dominant solvation environment are shown in Figure 4. It should be noted that the REMD results were found to be similar and the fractions of the majority species for each mixture from REMD simulations are shown in Figure S2 of the SI.

**Figure 4:**
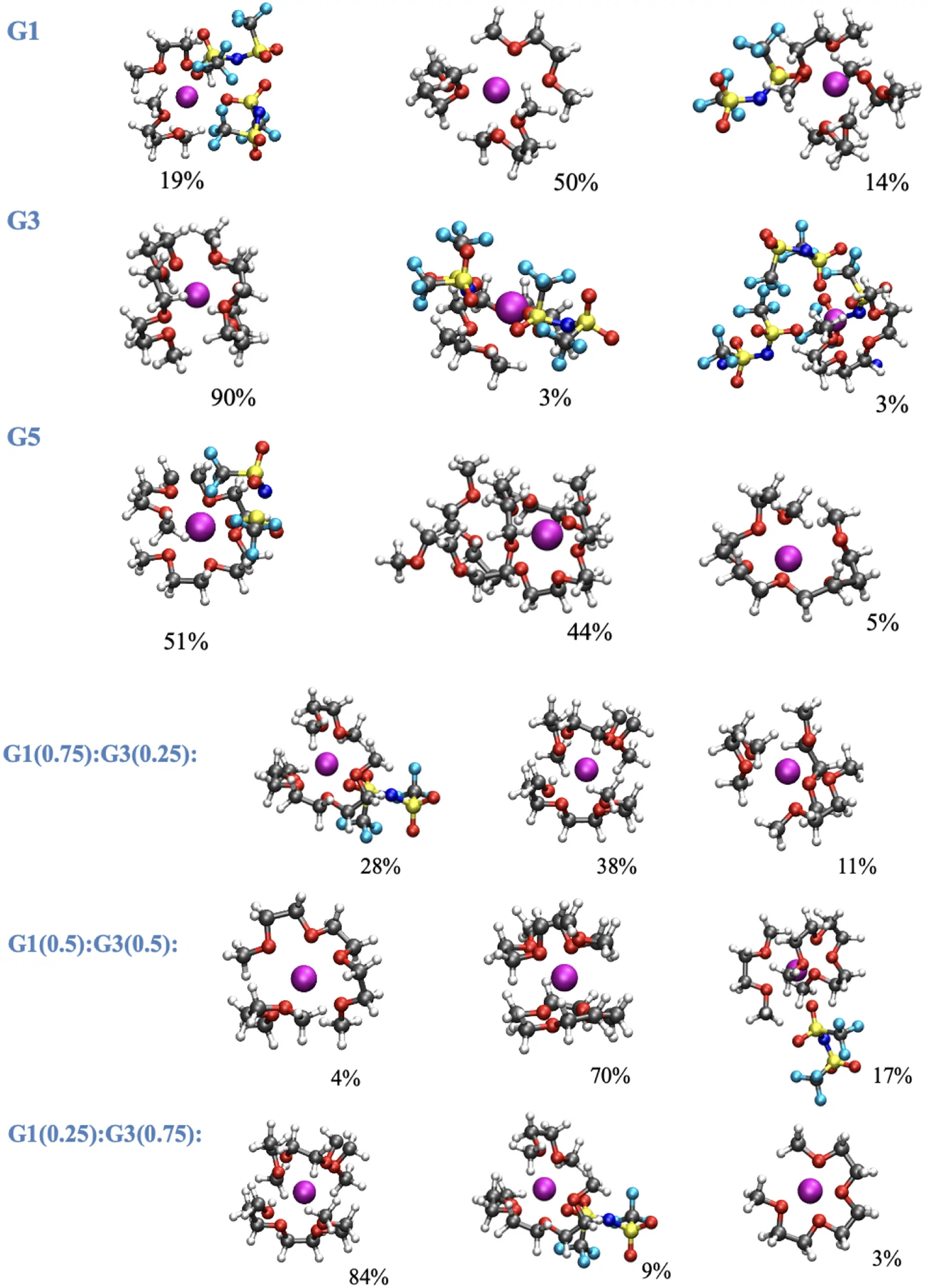
Snapshots of the major solvation structures (with their corresponding abundance percentages) for Na^+^ in six different glyme-based electrolyte systems with TFSI^–^ as the anion. The color scheme follows that used in Figure 1.

In pure G3, the solvation shell adopts a remarkably uniform configuration with 90% of Na^+^ coordinated by only two G3 molecules (2G3), which together provide the oxygen coordination required by Na^+^ (Figure 3). The 2G3 + 0TFSI environment accounts for 90% of all Na^+^ and effectively defines the structural character of the G3 system (Table S2). As G3 content decreases in the binary mixtures, the 2G3 fraction diminishes to 84% in G1(0.25):G3(0.75), 70% in G1(0.5):G3(0.5), and 38% in G1(0.75):G3(0.25), while the proportion of Na^+^ with zero or one G3 increases correspondingly. Importantly, even in G1(0.25):G3(0.75) where G3 constitutes 75% of the solvent, the 2G3 fraction remains 6% below that of pure G3, demonstrating that even a small amount of G1 is sufficient to partially disrupt the ordered 2G3 environment, and that the pure G3 solvation structure cannot be fully replicated in the binary mixture studied.

The solvation structure of Na^+^ in G5 is qualitatively distinct from all other systems studied in the present work, confirming that neither the number of chelating glyme oxygen atoms (G5 and the 0.5 binary mixture have the same number of chelating O atoms) nor glyme chain length is individually sufficient to predict solvation shell composition. In G5, Na^+^ exists in two nearly equally populated coordination states, with 56% coordinated by a single G5 molecule (1G5) and 44% by two G5 molecules (2G5). Unlike G1 and G3, which require multiple molecules to collectively satisfy the Na^+^ coordination requirement, G5 is the only glyme in this series capable of wrapping around a single Na^+^ and providing all six of its ether oxygens from a single chain. The bimodal distribution across 1G5/2G5 in pentaglyme stands in marked contrast to the structures seen in G1(0.5):G3(0.5), which despite providing the same ether oxygen-to-Na^+^ ratio, exhibits a fundamentally different structure characterized by a mixture of partial G1 and G3 coordination environments with a large population of free uncoordinated G1 molecules. The difference in structure between G5 and the mixture demonstrates that molecular architecture rather than the total number of available ether oxygen atoms governs solvation behavior.

The extent of TFSI^–^ participation in the Na^+^ first solvation shell is governed by the degree of glyme chelation and follows a clear trend across the series, though not a simple monotonic one (Figure 3, Table 1). In G1, where the Na^+^ solvation shell is assembled from multiple short-chain molecules, TFSI^–^ has substantial access to the coordination sphere, with 41% of Na^+^ having at least one TFSI^–^ in their first shell. Correspondingly, only 47% of TFSI^–^ remain free, while 13% act as bridging species simultaneously coordinating two or more Na^+^ (Table 1, Table S4), indicating significant ion association in G1. As G3 content increases across the mixture series toward pure G3, TFSI^–^ is progressively excluded from the Na^+^ coordination sphere, with the fraction of Na^+^ lacking any TFSI^–^ rising monotonically from 60% in G1(0.75):G3(0.25) to 77%, 88%, and 92% in G1(0.5):G3(0.5), G1(0.25):G3(0.75), and G3 respectively (Figure 3). In G3, the bridging TFSI^–^ population drops to 3%, and when TFSI^–^ enters the G3 solvation shell, it does so cooperatively, with 3% of Na^+^ coordinated by two TFSI^–^ and 4% by three or more, compared to only 0.4% with a single TFSI^–^, a pattern reflecting the selective and collective character of TFSI^–^ access to the tightly sealed 2G3 coordination environment (Table S2). G5 breaks this exclusion trend: 48% of Na^+^ in G5 have one TFSI^–^ in their first shell, and the fraction lacking TFSI^–^ drops to 52%, lower than even G1. The elevated TFSI^–^ participation in the pure G5 solution arises from the 1G5 coordination mode, in which a single G5 chain coordinates Na^+^, but leaves sites accessible for TFSI^–^. The sodium bridging TFSI^–^ population in G5 reaches 10%, substantially higher than in G3 and approaching the level observed in G1, indicating that despite its long chain length, G5 promotes more ion association than G3. Taken together, the TFSI^–^ coordination data reveal that ion association in these electrolytes is not a simple function of glyme chain length. Rather, it is governed by whether the glyme coordination environment is sufficient to exclude TFSI^–^ entirely. G3 provides complete solvation through multidentate chelation in the 2G3 mode, while G5, despite its longer chain, allows TFSI^–^ access through the incomplete coordination in 1G5 mode, making the two systems chemically distinct in how they regulate Na^+^–TFSI^–^ interaction.

**Table 1:** Fractions of TFSI^–^ Classified by the Number of Na^+^ Simultaneously Coordinated

System	Free TFSI^–^ (0 Na^+^)	TFSI^–^ with 1 Na^+^	TFSI^–^ with ≥ 2 Na^+^
**G1**	0.47	0.40	0.13
**G**1(0.75)**:G**3(0.25)	0.57	0.36	0.07
**G**1(0.5)**:G**3(0.5)	0.74	0.23	0.03
**G**1(0.25)**:G**3(0.75)	0.86	0.13	—
**G3**	0.82	0.15	0.03
**G5**	0.60	0.31	0.09

The solvation fraction analysis establishes two distinct classes of Na^+^ coordination environments in the binary mixtures: those dominated by G3 and those retaining G1, whose relative populations shift systematically with mixture composition. The structural picture established above raises fundamental questions about the spatial and temporal organization of Na^+^ relative to each other: how are these coordination environments arranged in the ionic network, and how do they evolve over time? The distinct van Hove correlation function *G_d_(r,t)* and the Na^+^–Na^+^ radial distribution function provide direct answers, revealing not only the static spatial organization of Na^+^, but the time scales over which that organization persists. The Na^+^–Na^+^ RDF (see Figure 5) exhibits two universal features shared across all six systems (peaks at approximately 8–9 Å and 12–13 Å) and one feature unique to G3 and the G3-containing mixtures (a sharp peak at approximately 3.5–4 Å). The universal peaks at 8–9 Å and 12–13 Å arise from the excluded volume of adjacent Na^+^ solvation shells and are present in all systems, with conditional pair analysis confirming that 92–99% of Na^+^ pairs at these distances share no molecular bridge of any kind (Table S5).

**Figure 5:**
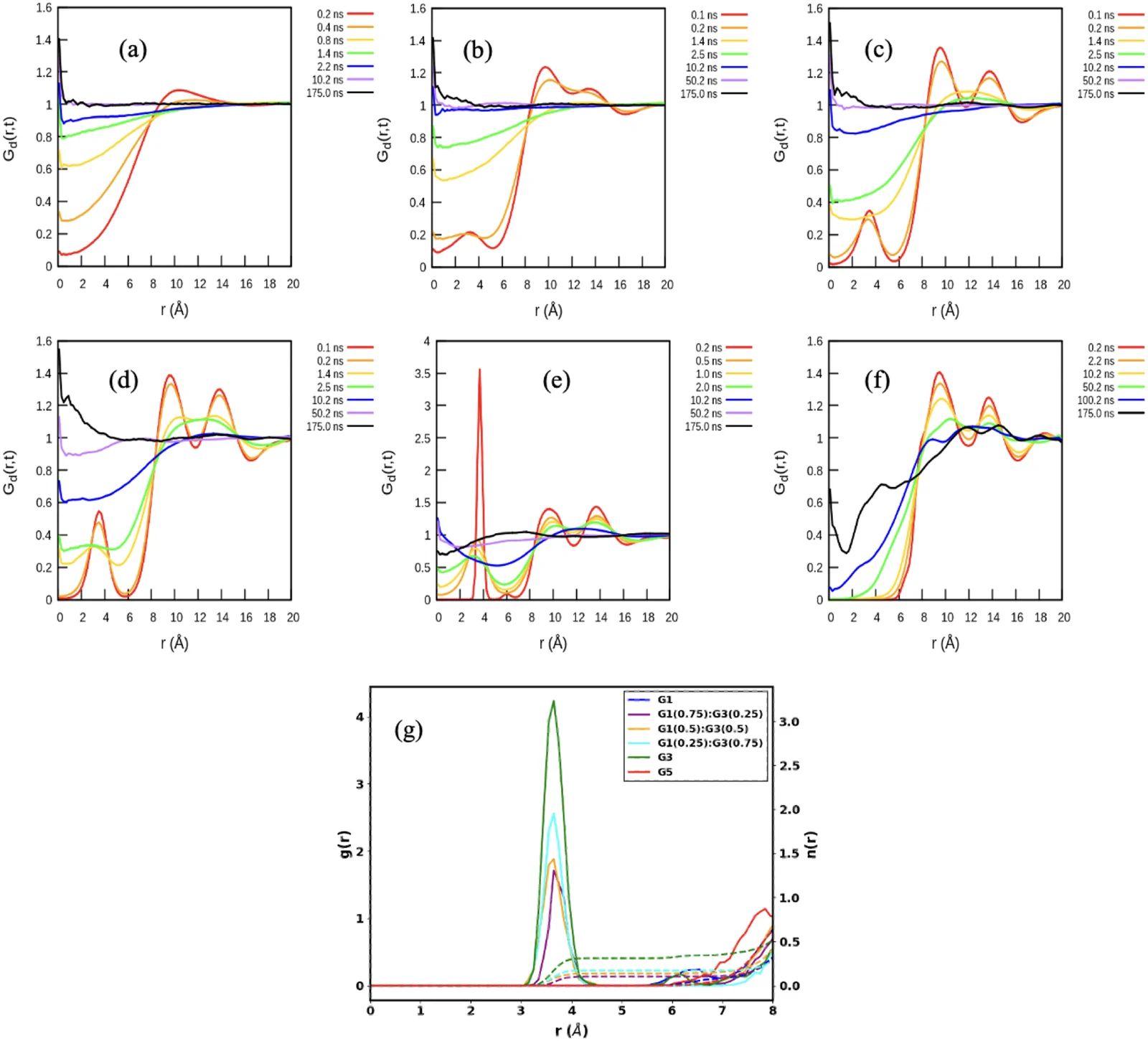
Distinct van Hove correlation functions, *G_d_(r, t)*, for NaTFSI–glyme electrolytes, corresponding to (a) G1, (b) G1(0.75):G3(0.25), (c) G1(0.5):G3(0.5), (d) G1(0.25):G3(0.75), (e) G3, and (f) G5. (g) Na^+^–Na^+^ radial distribution functions, *g*_Na–Na_(*r*) (solid lines), and coordination numbers, *n*(*r*) (dashed lines), for all six systems.

The peak at 3.5–4 Å in Figure 5 presents a fundamentally different origin. In G1, this feature is essentially absent, with the negligible population at this distance being exclusively TFSI-mediated as confirmed by conditional pair analysis (Table S5). In G5, no sodium pairs are found at this distance, as the large, excluded volume of G5 solvation shells physically prevents Na^+^ ions from approaching this closely. In G3, by contrast, a sharp and prominent peak with the largest peak amplitude in the Na^+^–Na^+^ RDF across the entire series is observed at 3.5–4 Å. Conditional pair analysis confirms that the pairs observed at this peak are mediated exclusively by glyme, establishing that this feature arises entirely from glyme-bridged Na^+^ contacts (Table S5). At these intercation distances, 37% of G3 molecules are simultaneously coordinating two Na^+^, directly confirming the bridging geometry (Table S4). This is confirmed further by the global glyme bridging analysis: around 5% of G3 molecules simultaneously coordinate two Na^+^ ions, compared to essentially none for G1 and G5 (Table 2). These Na^+^–G3–Na^+^ bridges arise from G3′s intermediate chain length: long enough that two oxygens coordinate one Na^+^ while the other two reach a second Na^+^, but not long enough to completely wrap around a single cation as G5 does. This bridge geometry fixes the two Na^+^ at approximately 3.5–4 Å separation, producing the sharp RDF peak unique to G3. Molecular snapshots confirm this structure directly (Figure 6).

**Table 2:** Fractions of Glyme Molecules Coordinating 0, 1, or 2 Na^+^ Simultaneously

System	Glyme-0Na	Glyme-1Na	Glyme-2Na
**G1**	0.54	0.46	—
**G**1(0.75)**:G**3(0.25)	0.56	0.43	—
**G**1(0.5)**:G**3(0.5)	0.51	0.47	0.01
**G**1(0.25)**:G**3(0.75)	0.44	0.54	0.02
**G3**	0.41	0.54	0.05
**G5**	0.27	0.73	—

**Figure 6:**
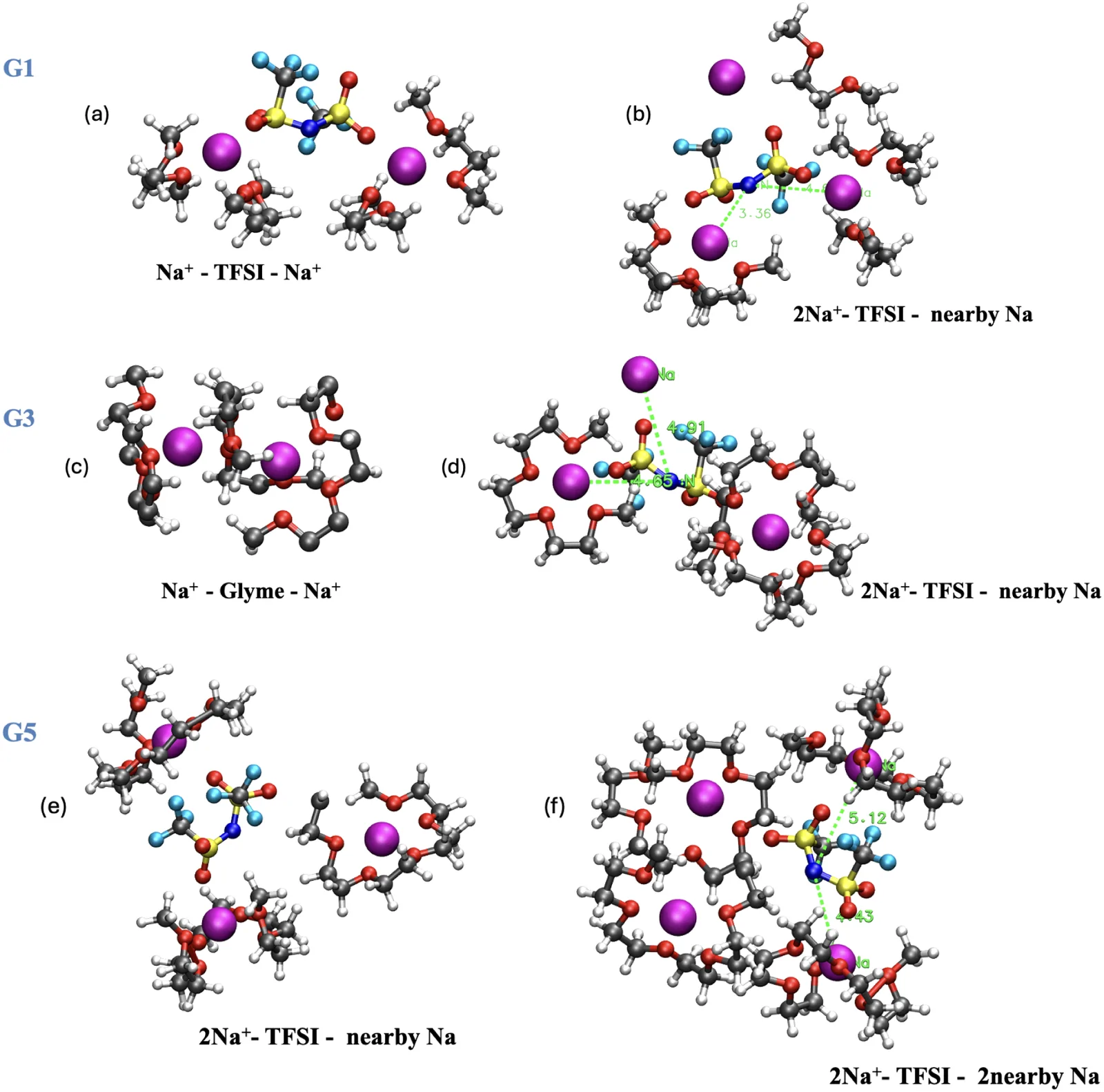
Representative simulation snapshots illustrating the dominant Na^+^ pair configurations identified from the conditional pair analysis for G1, G3, and G5. For G1: (a) Na^+^–TFSI^–^–Na^+^ bridging configuration and (b) a TFSI^–^-bridged Na^+^ pair with an additional nearby Na^+^. For G3: (c) the Na^+^–G3–Na^+^ glyme-bridged configuration characteristic of the 3.5–4 Å peak and (d) a TFSI^–^-bridged Na^+^ pair with an additional nearby Na^+^. For G5: (e) a TFSI^–^-bridged Na^+^ pair with one nearby Na^+^ and (f) a TFSI^–^-bridged Na^+^ pair with two nearby Na^+^. Na^+^ is shown in purple, carbon in gray, oxygen in red, hydrogen in white, sulfur in yellow, fluorine in cyan, and nitrogen in blue. Green dashed lines indicate Na^+^–N­(TFSI^–^) distances in Å.

The Na^+^–G3–Na^+^ glyme bridging geometry is not confined to pure G3 but persists in the Na^+^–Na^+^ structure in all three binary mixtures. The approximately 4 Å peak in the Na^+^–Na^+^ RDF is present in all mixtures, with peak amplitude increasing systematically with G3 content: *g­(r)* ≈ 1.73 in G1(0.75):G3(0.25), ≈1.88 in G1(0.5):G3(0.5), and ≈2.55 in G1(0.25):G3(0.75), compared to around 4.20 in pure G3 (Figure 5). The systematic increase in peak height directly reflects the growing population of 2G3-coordinated Na^+^ (i.e., G3 bridging), tracking the 2G3 fractions of 38%, 70%, and 84% across the three mixtures. Even in G1(0.75):G3(0.25), where G3 constitutes only 25% of the solvent, the G3 bridging geometry is already present and measurable in the Na^+^–Na^+^ pair structure, confirming that the G3 coordination environment extends to the pairwise organization of the ionic network.

The time evolution of *G_d_(r, t)* reveals how rapidly each system loses memory of the ion structural arrangements (Figure 5). In G1, all Na^+^–Na^+^ structural correlations decay to unity within approximately 2 ns, indicating rapid reorganization of the ionic network consistent with the loosely assembled and dynamically accessible G1 solvation environment. In the binary mixtures, the relaxation time scale increases progressively with G3 content. In G1(0.75):G3(0.25) the correlations relax within approximately 2–3 ns, similar to pure G1, reflecting the continued dominance of the G1 coordination environment. In G1(0.5):G3(0.5), significant structure persists to approximately 10 ns before disappearing, and in G1(0.25):G3(0.75), correlations persist to approximately 50 ns. Complete structural relaxation is achieved within the 200 ns simulation window for all four of these systems. The progressive slowing of the van Hove decay across the mixture series mirrors the progressive increase in the 2G3 fraction, confirming that the G3 coordination environment is directly responsible for the increasingly persistent Na^+^–Na^+^ structural correlations in the mixtures.

In G3, Na^+^–Na^+^ correlations persist on significantly longer time scales than in any of the mixtures, with *G_d_(r, t)* still showing residual structure at tens of nanoseconds and approaching unity only near 100 ns (Figure 5). Two distinct features characterize the G3 van Hove correlation function. The correlation peak at approximately 3.5–4 Å, visible at early times and decaying slowly over several nanoseconds, reflects the lifetime of the Na^+^–G3–Na^+^ bridged pairs confirmed above. The slow decay of this feature relative to the corresponding peak in the mixtures reflects the rigidity and stability of the 2G3 coordination environment: once a G3 molecule establishes a bridge between two Na^+^, both ions are held in place by their surrounding G3 framework, making the bridged configuration long-lived. The second peak at approximately 6–7 Å at early times in G3 corresponds to TFSI^–^-mediated correlation and decays relatively quickly, consistent with the rare and transient nature of TFSI^–^ coordination in this system.

G5 exhibits the most striking behavior in the series, with Na^+^–Na^+^ correlations persisting throughout the entire 200 ns trajectory and *G_d_(r, t)* not converging to unity within the simulation time scale (Figure 5). Unlike all other systems, G5 does not show a Na^+^–Na^+^ peak below 6–7 Å, consistent with the large physical barrier imposed by the G5 solvation shells. The persistent correlations in G5 arise from the slow collective dynamics of the long G5 chains themselves, particularly in the 2G5 coordination mode where two G5 chains must simultaneously reorganize for structural relaxation to occur. The origin of the persistent correlations in G5 is mechanistically distinct from G3, where the slow dynamics reflect the stability of the glyme-bridged Na^+^ pairs, and from G1, where rapid reorganization reflects the mobility of the loosely assembled solvation environment. The TFSI^–^-mediated correlation at approximately 6–7 Å is also present in G5 and persists longer than in any other system, consistent with the collective character of TFSI^–^ bridging in G5 where bridging TFSI^–^ molecules see a local ionic environment with multiple nearby Na^+^ (Table S3). Together, these observations confirm that G5 operates in a qualitatively distinct dynamic regime from all other systems, one in which the slow collective reorganization of the solvation shell itself determines the persistence of Na^+^–Na^+^ structural correlations. The structural differences described above directly influence the transport behavior of the electrolyte systems.

### 3.2 Dynamic Properties

The self-diffusion coefficients of Na^+^ and TFSI^–^ decrease systematically across the series, spanning nearly 3 orders of magnitude from G1 to G5 (Figure 7, Table 3). In G1, both ions exhibit the highest mobility, with Na^+^ and TFSI^–^ diffusion coefficients of 1.8 and 2.0 × 10^–10^ m^2^/s, respectively. Across all systems, TFSI^–^ diffuses consistently faster than Na^+^, reflecting the weaker coordination environment of the anion relative to the strongly chelated Na^+^ solvation shell. As G3 content increases across the binary mixtures, diffusion coefficients decrease continuously, reaching 6.1 and 7.8 × 10^–12^ m^2^/s for Na^+^ and TFSI^–^ in pure G3, and 5.0 and 6.2 × 10^–13^ m^2^/s in pure G5. The mean squared displacement curves confirm fully diffusive behavior for Na^+^ and TFSI^–^ in G1, G3, and the three binary mixtures within the simulation time scale. G5 is the exception, showing subdiffusive dynamics that do not change within the accessible simulation window (Figure S4).

**Figure 7:**
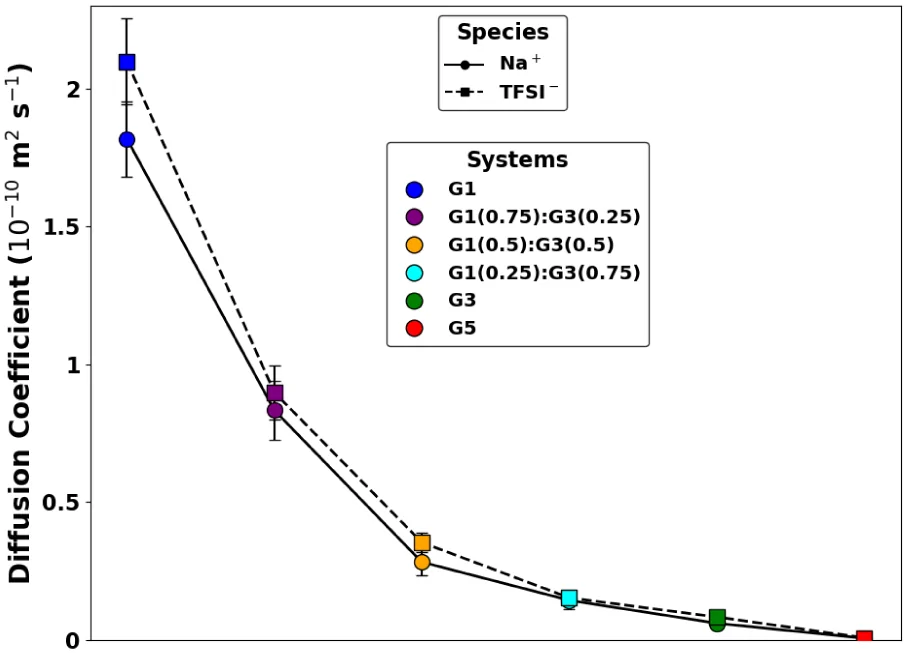
Diffusion coefficients of Na^+^ (solid lines) and TFSI^–^ (dashed lines) ions in each electrolyte system.

**Table 3:** Diffusion Coefficient Decomposition for Binary G1:G3 Mixtures^a^

System	G1 fraction	G3 fraction	D_predicted_ (× 10^–11^ m^2^/s)	D_actual_ (× 10^–11^ m^2^/s)
**G**1(0.75)**:G**3(0.25)	0.58	0.42	10.8	8.5
**G**1(0.5)**:G**3(0.5)	0.25	0.75	5.1	2.8
**G**1(0.25)**:G**3(0.75)	0.11	0.90	2.5	1.4

a Predicted values were obtained by weighting the pure component Na^+^ diffusion coefficients of G1 (1.8 × 10^–10^ m^2^/s) and G3 (6.1 × 10^–12^ m^2^/s) by the fraction of Na^+^ coordinated by each glyme type as determined from the solvation shell fraction analysis.

The diffusion behavior of the binary mixtures is directly governed by their solvation environments. Because G3 preferentially occupies the Na^+^ first solvation shell, even at low concentrations, the mixture contains two distinct populations of Na^+^: those coordinated primarily by G3, which diffuse at near-G3 rates, and those retaining G1 coordination, which diffuse faster at near-G1 rates. To test this interpretation quantitatively, the Na^+^ diffusion coefficient of each mixture was estimated by weighting the pure component diffusion coefficients by the fraction of Na^+^ coordinated by each glyme type, using the solvation fractions established in Figure 3. The results, summarized in Table 3, show that for G1(0.75):G3(0.25), where 58% of Na^+^ retain G1 coordination and 42% are G3 coordinated, the predicted diffusion coefficient is 10.8 × 10^–11^ m^2^/s compared to the simulated value of 8.5 × 10^–11^ m^2^/s. For G1(0.5):G3(0.5), the prediction yields 5.1 × 10^–11^ m^2^/s against 2.8 × 10^–11^ m^2^/s, and for G1(0.25):G3(0.75), the prediction yields 2.5 × 10^–11^ m^2^/s compared to 1.4 × 10^–11^ m^2^/s. Hence, the predicted values match surprisingly well with the simulated values, confirming that the mixture dynamics reflect the presence of two distinct populations rather than a homogeneous averaged environment.

A direct consequence of this finding is that the presence of free G1 in the mixtures, while reducing viscosity, does not generate additional conductivity beyond what is accounted for by the individual diffusing populations. The Nernst–Einstein conductivity, which depends solely on self-diffusion coefficients, decreases monotonically across the series from 1.85 S/m in G1 to 0.07 S/m in G3 and 0.04 S/m in G5, consistent with the diffusion trends (Figure 8). The NEMD conductivity follows a similar trend and shows good agreement with experimental values for G1 and the G1-rich mixtures, validating the simulation approach for these systems. The largest discrepancy between simulated and experimental conductivity occurs in G5, where the simulated conductivity falls substantially below the experimental value. The gap between experiment and simulations for G5 is a direct consequence of the persistent Na^+^–Na^+^ correlations and slow conformational dynamics of the G5 chains, which require time scales well beyond 200 ns to fully relax as evidenced by the system’s subdiffusive behavior within the simulated time window. Furthermore, classical nonpolarizable force fields are known to underestimate transport properties for viscous electrolyte systems,^73,74^ and in addition, full convergence may require simulation time scales on the order of microseconds.^74^ The ion association in pure glyme-based sodium electrolytes for G1 to G4 predicted from simulations with the same force-field as was used in this work matched, quite well, with the experimental data from FTIR spectroscopy, while the diffusion constants, from pulse field gradient NMR experiments, of the glyme molecules in these electrolytes was found to be underestimated by almost a factor of 5 for G4, but was significantly closer to experiment for shorter chained glymes.^40^ Therefore, while the conductivity of the G5 electrolyte is much lower as compared to experiment, due to the underestimation of the glyme solvent diffusion, these simulations should predict the correct solvation structures and ion association trends. The use of polarizable force fields may help bridge the discrepancy in transport properties.^73,75^

**Figure 8:**
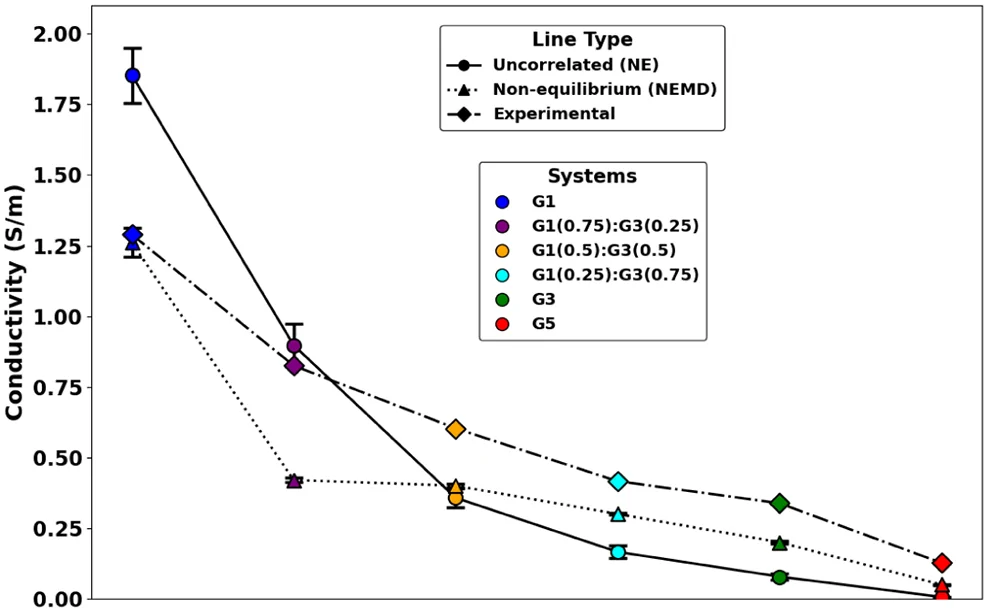
Ionic conductivity (S/m) of NaTFSI-based glyme electrolytes comparing uncorrelated (Nernst–Einstein), nonequilibrium, and experimental values.

For G3-rich systems, the NEMD conductivity is higher than the Nernst–Einstein value whereas for the G1-rich systems it is lower than the uncorrelated Nernst–Einstein values, implying that conductivity is dependent on more than simply the uncorrelated diffusion of ions. The cation–anion cross-term λ_+–_ reveals how cation–anion association evolves across the series (Table 4, Figure S3). The cross-term is negative in G1 and G1(0.75):G3(0.25), reflecting correlated Na^+^–TFSI^–^ motion arising from the significant ion pairing and bridging TFSI^–^ population, as discussed above. The most negative cation–anion cross-term occurs in G1(0.75):G3(0.25), where the partial displacement of G1 by G3 leaves Na^+^ in a transitional coordination state that is neither fully G1-coordinated nor fully wrapped by G3, creating conditions most favorable for Na^+^–TFSI^–^ ion pairing across the entire series. As G3 content increases and TFSI^–^ is progressively excluded from the Na^+^ solvation shell, the cross-term shifts from near-zero in G1(0.5):G3(0.5) to positive in G1(0.25):G3(0.75). The G1(0.25):G3(0.75) electrolyte is the only mixture to exhibit a positive cross-term (λ_+–_ = +0.07 S/m), reflecting anticorrelated Na^+^–TFSI^–^ motion that contributes favorably to net charge transport. This behavior connects directly to the solvation structure of the mixture: with 84% of Na^+^ in the 2G3 + 0TFSI environment with TFSI^–^ almost entirely excluded from the Na^+^ coordination sphere and thereby losing the correlated ion-pair motion that reduced conductivity in G1-rich systems. At the same time, the remaining 11% of Na^+^ retaining G1 coordination maintains sufficient fluidity for anticorrelated motion to generate a positive contribution to conductivity. Pure G3 has the TFSI^–^ exclusion, but lacks the fluidity provided by free G1, yielding a near-zero cross-term. In addition, the G3 NEMD conductivity is higher than the Nernst–Einstein uncorrelated value. At first this seems surprising since the anticorrelated cation–anion contribution is small but slightly negative. However, the slower decay of Na^+^–Na^+^ peaks in the distinct van Hove correlation plots for the G3 case point to the larger nonself cation–cation correlations that contribute to this increase compared to the uncorrelated conductivity. In G5, the cross-term is negligibly small, reflecting the strongly restricted ion motion associated with the slowly reorganizing G5 solvation shells. These results confirm that G1(0.25):G3(0.75) achieves the optimal balance between TFSI^–^ exclusion and retained ionic fluidity among all systems studied.

**Table 4:** Cation–Anion Correlated Conductivity (Cross-Term λ_+–_) for All Systems

System	λ_+–_(S/m)
**G1**	–0.12
**G**1(0.75)**:G**3(0.25)	–0.16
**G**1(0.5)**:G**3(0.5)	0.00
**G**1(0.25)**:G**3(0.75)	+0.07
**G3**	–0.01
**G5**	+0.001

The shear viscosity increases monotonically from G1 to G5 (Figure 9). While the progressively more stable Na^+^ coordination environments identified in the solvation structure analysis may contribute to the increase, the trend is also consistent with the intrinsically higher viscosity of longer-chain glymes independent of ion coordination. The MD viscosities show good agreement with experimental values for G1 and the binary mixtures. In G3, the simulated viscosity is higher than the experimental value, consistent with the highly stable 2G3 coordination environment identified in the solvation structure analysis, which restricts molecular motion more than what is observed experimentally. In G5, the simulated viscosity agrees well with experiment, confirming that the short-time scale dynamics are captured accurately by the simulation.

**Figure 9:**
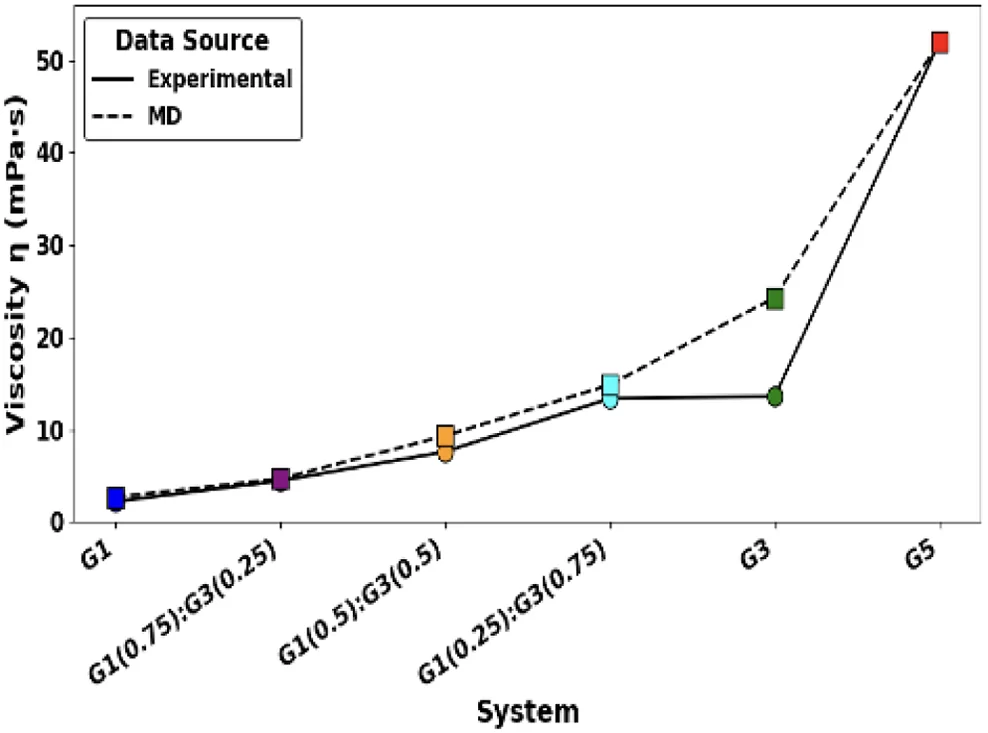
Viscosity of NaTFSI in G1, G3, G5, and binary G1:G3 mixtures obtained from MD simulations and experimental measurements.

The Walden analysis connects ionic conductivity and viscosity into a single measure of transport efficiency across the series (Table 5). All systems fall below the ideal Walden line, confirming that ion motion is influenced by ion–ion correlations throughout the series. Experimentally, G1(0.25):G3(0.75) exhibits the smallest Walden deviation among the binary mixtures (ΔW_EXP_ = 0.40, ΔW_MD_ = 0.52), reflecting the most efficient ion transport. This result is consistent with the positive λ_+–_, the near-complete TFSI^–^ exclusion, and the residual G1 fluidity described above, all of which combine to make G1(0.25):G3(0.75) the optimal composition among the systems studied. Pure G3 achieves strong TFSI^–^ exclusion, but its higher viscosity and lack of free G1 result in a slightly larger Walden deviation (ΔW_EXP_ = 0.43, ΔW_MD_ = 0.56) than G1(0.25):G3(0.75), confirming that TFSI^–^ exclusion alone is insufficient and that the balance between exclusion and fluidity is critical. The close agreement with experiment demonstrates that the force field model is well suited for investigating solvation structure and transport trends across the glyme series, while highlighting that extended simulation time scales are required to fully resolve the slow conformational dynamics of G5 and accurately reproduce its experimental conductivity.

**Table 5:** Walden Deviations Δw from Experimental (EXP) and Simulations (MD) for G1, G3, and the Binary G1:G3 Mixtures

System	ΔW_EXP_	ΔW_MD_
**G1**	0.65	0.60
**G**1(0.75)**:G**3(0.25)	0.56	0.83
**G**1(0.5)**:G**3(0.5)	0.48	0.59
**G**1(0.25)**:G**3(0.75)	0.40	0.52
**G3**	0.43	0.56

Unlike self-diffusion, which can be tuned predictably by adjusting the solvation fractions through composition, conductivity depends additionally on the correlated motion between distinct cation–cation (nonself), distinct anion–anion (nonself) and cation–anion species. The self-ion correlations are captured by the self-diffusion. The distinct cation–cation van Hove correlations decay relatively fast in time, except for the G5 case. Therefore, these correlations will not contribute greatly to the conductivity. The large fraction of free TFSI anions also results in relatively small contributions to distinct TFSI–TFSI correlated motions, except for the G3 case. Further, the contributions of cation–anion correlation to conductivity, captured by 2λ_+–_ (the 2 reflections +– and −+ contributions), are not always negligible (see Table 4). When the cation and anion motions are anticorrelated, the motion contributes positively to the conductivity and hence 2λ_+–_ is positive. On the other hand, if the cation solvation shell has two or more negative anions, this results in a solvated cation with net negative charge, thereby reducing the conductivity and contributing to the negative value of 2λ_+–_. The G1 case has almost 20% of cations in this type of coordination and hence shows a negative 2λ_+–_ contribution to conductivity. In the case where there is a single anion in the cation solvation shell, the species has a net zero charge but anion hopping from the shell can also contribute to anticorrelated motion, thereby contributing positively to the 2λ_+–_ term. Thus, solvation structure and hopping can both affect the conductivity through correlated motions of the cation and anion.

To examine how frequently TFSI^–^ exchanges between Na^+^, the hopping function *H_i_* (*t*),^40^ was computed over the trajectories (Table S6). The net hopping rate decreases by more than 2 orders of magnitude across the series, from 7.2 × 10^–4^ ns^–1^ Å^–3^ in G1 to 3.6 × 10^–6^ ns^–1^ Å^–3^ in G3, tracking the progressive exclusion of TFSI^–^ from the Na^+^ solvation shell. The hopping and conductivity results together with the solvation structures reveal that a positive λ_+–_ requires two conditions to be satisfied simultaneously. The first condition is structural: there should be a significantly large fraction of TFSI^–^ excluded from the Na^+^ coordination sphere, achieved by the 2G3 chelation environment, while still maintaining a non-negligible fraction of Na^+^ with just one TFSI^–^ in its solvation shell. The second condition is mobility-related: the medium must remain fluid enough for anticorrelated Na^+^–TFSI^–^ motion to develop the positive contribution to λ_+–_. This is achieved by the presence of free G1 in the bulk liquid after G3 preferentially occupies the Na^+^ solvation shells, preserving sufficient fluidity despite the persistence of the G3-dominated coordination environment. The G1-rich systems fail the first condition: with approximately 40% of Na^+^ coordinating TFSI^–^ and high hopping rates reflecting pairs that continuously form, comigrate, and reform with new partners, the negative contribution dominates and λ_+–_ remains negative. Pure G3 satisfies the 2G3 solvation part of the first condition more strongly than the mixtures, but the near-complete encapsulation of Na^+^ by two G3 molecules leaves only a small fraction of Na^+^ with a single TFSI^–^ in their shell, and the absence of free G1 removes the fluidity needed for the anticorrelated contribution to develop, leaving λ_+–_ negligible. The hopping results confirm this essentially persistent ionic environment, with rare hopping and 48% of the Na^+^ that coordinate a TFSI^–^ retaining the same anion throughout the simulation. G5 also fails the second condition: TFSI^–^ leaves and returns to the same Na^+^ at nearly equal rates (0.13 and 0.12 ns^–1^), oscillating within the same coordination shell without net exchange, and the slow dynamics of the G5 chains render the resulting displacements negligible, again leaving λ_+–_ ≈ 0.00. Only G1(0.25):G3(0.75) satisfies both conditions: 84% of Na^+^ are coordinated by two G3 molecules without TFSI^–^ participation in the first solvation shell, while the remaining Na^+^ maintain a small but real population with a single TFSI^–^, and the residual free G1 population preserves sufficient bulk mobility despite the persistent G3-dominated solvation shells. The character of the hopping in this system further distinguishes it from G1. G1(0.25):G3(0.75) has the lowest fractions of 1Na and 2Na TFSI^–^ coordination among all systems (Table 1), meaning contact pairs and bridging aggregates are nearly absent. The rare hops that do occur originate predominantly from Na^+^ coordinating a single TFSI^–^ (Na-1TFSI species) in a nonaggregate environment, and when TFSI^–^ departs from such an environment, Na^+^ and TFSI^–^ move in opposing directions, generating the anticorrelated displacement that produces the positive λ_+–_ (Tables S7 and S10). In G1, by contrast, TFSI^–^ anions show the highest fractions of 1Na and 2Na coordination among all systems (Table 1), forming contact pairs and bridging aggregates that dominate the hopping events. Despite the large total hopping rate, the motion of TFSI^–^ between Na^+^ within these aggregates remains correlated with the surrounding ionic cluster, contributing negatively to λ_+–_. This persistence is confirmed directly by the glyme coordination analysis, which does not show complete exchange of the Na^+^ glyme shell for any Na^+^ in this system over the entire trajectory, in contrast to G1 where the more labile G1 shells undergo frequent exchange (Tables S8 and S9). This is why G1(0.25):G3(0.75) alone exhibits a positive λ_+–_ (contributing around 25% of the total NEMD conductivity), the smallest Walden deviation, and the most efficient charge transport per unit fluidity in the series. The balance of these two conditions also explains why diffusion and conductivity respond differently to composition: diffusion depends only on how fast individual ions move and is therefore tunable through the solvation fractions, whereas conductivity depends on the relationship between cation and anion motion, which only specific compositions achieve.

## 4 Conclusion

This study has examined how glyme chain length and molecular architecture govern the solvation structure and transport properties of NaTFSI-based electrolytes across pure G1, G3, and G5 and three binary G1:G3 mixtures. The central structural finding is that molecular architecture, rather than the average number of coordinated ether oxygen atoms, determines electrolyte behavior. This is demonstrated most clearly by G5, which despite providing the same oxygen-to-Na^+^ ratio as G1(0.5):G3(0.5) adopts a continuous single-chain coordination that produces a uniquely slow dynamic regime, with Na^+^–Na^+^ correlations that persist throughout the entire simulation and subdiffusive behavior seen throughout the simulation time frame. The same principle underlies the preferential displacement of G1 by G3, which generates the free G1 population that sustains fluidity in the G1-rich systems, and the rigid 2G3 chelation that excludes TFSI^–^ and minimizes ion pairing in G3-rich systems.

The transport analysis reveals the importance of cation–anion correlations, which changes nonmonotonically as a function of composition, on the conductivity. The cation–anion correlated motion that impacts conductivity requires two competing conditions to be satisfied at once: TFSI^–^ must be excluded from the Na^+^ coordination sphere to suppress the correlated motion of contact ion pairs but there should still exist a non-negligible fraction of Na^+^ with a single TFSI^–^ in the solvation shell, while the medium must remain fluid enough for the ion motion that contributes positively to charge transport. The TFSI^–^ hopping and glyme shell exchange analyses showed that these conditions are met simultaneously only in G1(0.25):G3(0.75), which alone exhibits a positive cross-term and the smallest experimental Walden deviation in the series. G1-rich systems retain too much TFSI^–^ in the Na^+^ shell, while pure G3 and G5 immobilize the ions so completely that the necessary motions are absent.

These results establish that the efficient transport of glyme-based sodium electrolytes cannot be optimized through a single structural parameter but requires the simultaneous control of ion exclusion and solvent fluidity, properties that can be tuned independently through the choice and proportion of glyme solvents. This design rule provides a concrete basis for engineering mixed-glyme electrolytes and motivates further study of whether the same balance can be achieved with other salts, anions, or at higher concentrations, where the interplay between solvation architecture and ion correlation may differ. The principal limitation of the present work concerns G5, whose slow conformational dynamics require extended simulation time scales to fully capture its experimental conductivity.

## Supplementary Material



## Data Availability

The data that support the findings of this study are available from the corresponding author upon reasonable request.

## Notes

Graphs comparing the radial distribution functions for monoglyme from AIMD with force-field simulations, temperature evolution in the REMD simulations, RDFs and solvation structure distributions from REMD simulations, the various terms of the correlated conductivity, and the mean squared displacements for the cation and anion. The tables include the composition for each simulation, the analysis of the different solvation environments around the sodium cation, the anion hopping rates, the glyme exchange rate, glyme identity change and the species resolved hopping rates (PDF)
