# Supplementary material for: Solvation Structure, Ion Association, and Transport in Sodium Glyme Electrolytes: From Monoglyme to Pentaglyme and Binary Mixtures
Source: J Phys Chem B. 2026 Sep 23;130(40):10674–89. doi: 10.1021/acs.jpcb.6c05191 (PMC13656733; doi:10.1021/acs.jpcb.6c05191)
Supplement: Supplementary file 1 [file jp6c05191_si_001.pdf]

Solvation Structure, Ion Association, and Transport in Sodium Glyme Electrolytes: From Monoglyme to Pentaglyme and Binary Mixtures

Blessing Adeleye,[1] Valeria Bonilla,[1] Orlando Carrillo-Bohórquez,[1] Fedra Leonik[1], Ryan Jorn,[,2] Daniel Kuroda*[1] and Revati Kumar*[1]*

[1]Department of Chemistry, Louisiana State University, 232 Choppin Hall, Baton Rouge, Louisiana 70803, USA

[2]Department of Chemistry, Wichita State University, 206 McKinley Hall, Wichita, KS 67260-0051, USA

*email: revatik@lsu.edu; dkuroda@lsu.edu; ryan.jorn@wichita.edu

SUPPORTING INFORMATION

Table S1. Composition of all six simulated systems showing the number of NaTFSI ion pairs, number of glyme molecules of each type, and total glyme ether oxygen atoms. In all cases, the number of NaTFSI ion pairs is 58 and the total number of glyme oxygen atoms is 696.

System	#G1	#G3	#G5
**G1**	348	—	—
**G3**	—	174	—
**G5**	—	—	116
**G1(0.75):G3(0.25)**	208	70	—
**G1(0.5):G3(0.5)**	116	116	—
**G1(0.25):G3(0.75)**	50	149	—

Table S2. Percentage of Na^+ ions within the indicated first-shell coordination environment. The table covers the pure G1 and G3 systems, and binary G1:G3 mixtures. Only environments with ≥1% of the population in at least one system are shown.

#G1 - #G3	#TFSI	G1 (%)	G1(0.75): G3(0.25) (%)	G1(0.5): G3(0.5) (%)	G1(0.25): G3(0.75) (%)	G3 (%)
**0 - 1**	0	—	2	2	3	3
**0 - 1**	1	—	1	1	1	—
**0 - 1**	2	—	1	1	2	3
**0 - 1**	≥3	—	—	1	—	3

**0 - 2**	0	—	38	70	84	90
**1 - 0**	1	1	1	—	—	—
**1 - 1**	0	—	7	4	2	—
**1 - 1**	1	—	28	17	9	—
**2 - 0**	0	8	2	—	—	—
**2 - 0**	1	6	1	—	—	—
**2 - 0**	2	19	4	2	—	—
**≥3 - 0**	0	50	11	1	—	—
**≥3 - 0**	1	14	3	—	—	—

Table S3. Percentage of Na^+ ions within the indicated first-shell coordination environment for the G5 system. Only environments with ≥1% of the population in at least one system are shown.

#G5	#TFSI	G5 (%)
**1**	0	5
**1**	1	51
**2**	0	44

Table S4. Distribution of $TFSI^-$-centered Na^+ coordination categories associated with selected Na^+–Na^+ distance windows for G1, G3, and G5. Na^+–Na^+ pairs were first identified within each specified distance window. $TFSI^-$ molecules satisfying the Direct (Na–N ≤ 5.5 Å) and Nearby (5.5 < Å Na–N ≤ 7.0 Å) selection criteria for the selected Na^+–Na^+ pairs were then identified and classified according to the number of directly coordinated and nearby Na^+ ions surrounding each $TFSI^-$. Population fraction (%) represents the percentage of all selected $TFSI^-$ observations across all distance windows, where the values add to 100% for each system. The remaining columns report the average distribution of the selected $TFSI^-$ observations among the four coordination categories, where 1Na Direct + 1 Nearby indicates one directly coordinated Na^+ and one additional nearby Na^+, 1Na Direct + 2+ Nearby indicates one directly coordinated Na^+ and two or more additional nearby Na^+ ions, 2+Na Direct Only indicates two or more directly coordinated Na^+ ions with no additional nearby Na^+ ions, and 2+Na Direct + Nearby indicates two or more directly coordinated Na^+ ions together with one or more additional nearby Na^+ ions.

Distance (Å)	System	Population Fraction (%)	1Na Direct + 1 Nearby	1Na Direct + 2+ Nearby	2+Na Direct Only	2+Na Direct + Nearby
**3.5–4.5**	**G1**	—	—	—	—	—
	**G3**	35	0.38	0.15	—	0.47
	**G5**	—	—	—	—	—
**6.5–7.5**	**G1**	20	0.17	0.06	0.55	0.23
	**G3**	15	0.15	0.15	0.09	0.61
	**G5**	28	0.17	0.07	0.45	0.30
**8.5–9.5**	**G1**	60	0.42	0.09	0.31	0.18
	**G3**	32	0.49	0.26	0.07	0.19
	**G5**	47	0.43	0.12	0.16	0.29
**10.5–11.5**	**G1**	20	0.60	0.18	—	0.22
	**G3**	18	0.57	0.23	—	0.20
	**G5**	25	0.48	0.14	—	0.37

Table S5. Distribution of glyme-centered Na^+ coordination categories associated with selected Na^+–Na^+ distance windows for G1, G3, and G5. Na^+–Na^+ pairs were first identified within each specified distance window. Glyme molecules coordinated to either Na^+ in the selected pairs using a Na–O(glyme) cutoff were then identified and classified according to the number of coordinated Na^+ ions surrounding each glyme molecule. Population fraction (%) represents the percentage of all selected glyme observations across all distance windows, where the values add to 100% for each system. The remaining columns report the average distribution of the selected glyme observations among the coordination categories, where Glyme-1Na indicates glyme molecules coordinated to one Na^+ and Glyme-2Na indicates glyme molecules coordinated to two Na^+ ions.

Distance (Å)	System	Population Fraction (%)	Glyme-1Na	Glyme-2Na
**3.5–4.5**	**G1**	—	—	—
	**G3**	7	0.63	0.37
	**G5**	—	—	—
**6.5–7.5**	**G1**	4	1.00	—
	**G3**	1	0.75	0.25
	**G5**	5	1.00	—
**8.5–9.5**	**G1**	47	1.00	—
	**G3**	42	0.89	0.11
	**G5**	42	1.00	—
**10.5–11.5**	**G1**	49	1.00	—
	**G3**	50	0.85	0.15
	**G5**	53	1.00	—

Table S6. Pair-centered Na^+–Na^+ peak sharing analysis showing the fraction of Na^+–Na^+ pairs at each distance window mediated by $TFSI^-$ only, glyme only, or neither, for G1, G3, and G5.

Distance (Å)	System	TFSI only	Glyme only	Neither
**3.5–4.5**	**G1**	1.00	—	—
	**G3**	—	1.00	—
	**G5**	—	—	—
**6.5–7.5**	**G1**	0.53	—	0.47
	**G3**	0.18	—	0.82
	**G5**	0.45	—	0.55
**8.5–9.5**	**G1**	0.08	—	0.92
	**G3**	0.01	—	0.99
	**G5**	0.03	—	0.97

Table S7. Net $TFSI^-$ hopping rate for all six systems.

System	Net Hopping Rate (ns^{-1} $Å^{-3}$)
**G1**	7.26×10^{-4}
**G1(0.75):G3(0.25)**	2.35×10^{-4}
**G1(0.5):G3(0.5)**	4.54×10^{-5}
**G1(0.25):G3(0.75)**	1.22×10^{-5}
**G3**	3.61×10^{-6}
**G5**	6.84×10^{-6}

Table S8. Glyme shell exchange rate per Na^+ and the fraction of Na^+ retaining or exchanging their glyme shell for all six systems.

System	Exchange Rate per Na^+ (ns^{-1})	Na^+ Retained Same Shell (%)	Na^+ Exchanged at Least Once (%)
**G1**	1.43×10^{-2}	7	93
**G1(0.75):G3(0.25)**	3.02×10^{-3}	78	22
**G1(0.5):G3(0.5)**	1.72×10^{-4}	97	3
**G1(0.25):G3(0.75)**	—	100	—
**G3**	1.72×10^{-4}	97	3
**G5**	—	100	—

Table S9. Glyme identity breakdown of complete shell exchanges in the binary G1:G3 mixtures. Values are counted with percentages in parentheses.

System	G1→G1	G3→G3	G1→G3	G3→G1	Mixed
**G1(0.75):G3(0.25)**	32 (91%)	—	—	—	3 (9%)
**G1(0.5):G3(0.5)**	1 (50%)	—	—	1 (50%)	—
**G1(0.25):G3(0.75)**	—	—	—	—	—

Table S10. Species-resolved hopping showing the percentage of Na^+ ions that undergo hopping in the Na-1TFSI$^-$ and Na-2TFSI$^-$ coordination environments for all six systems.

System	Percent that hop (Na-1TFSI$^-$)	Percent that hop (Na-2TFSI$^-$)
**G1**	18	26
**G1(0.75):G3(0.25)**	37	8
**G1(0.5):G3(0.5)**	21	3
**G1(0.25):G3(0.75)**	11	2
**G3**	—	2
**G5**	54	—

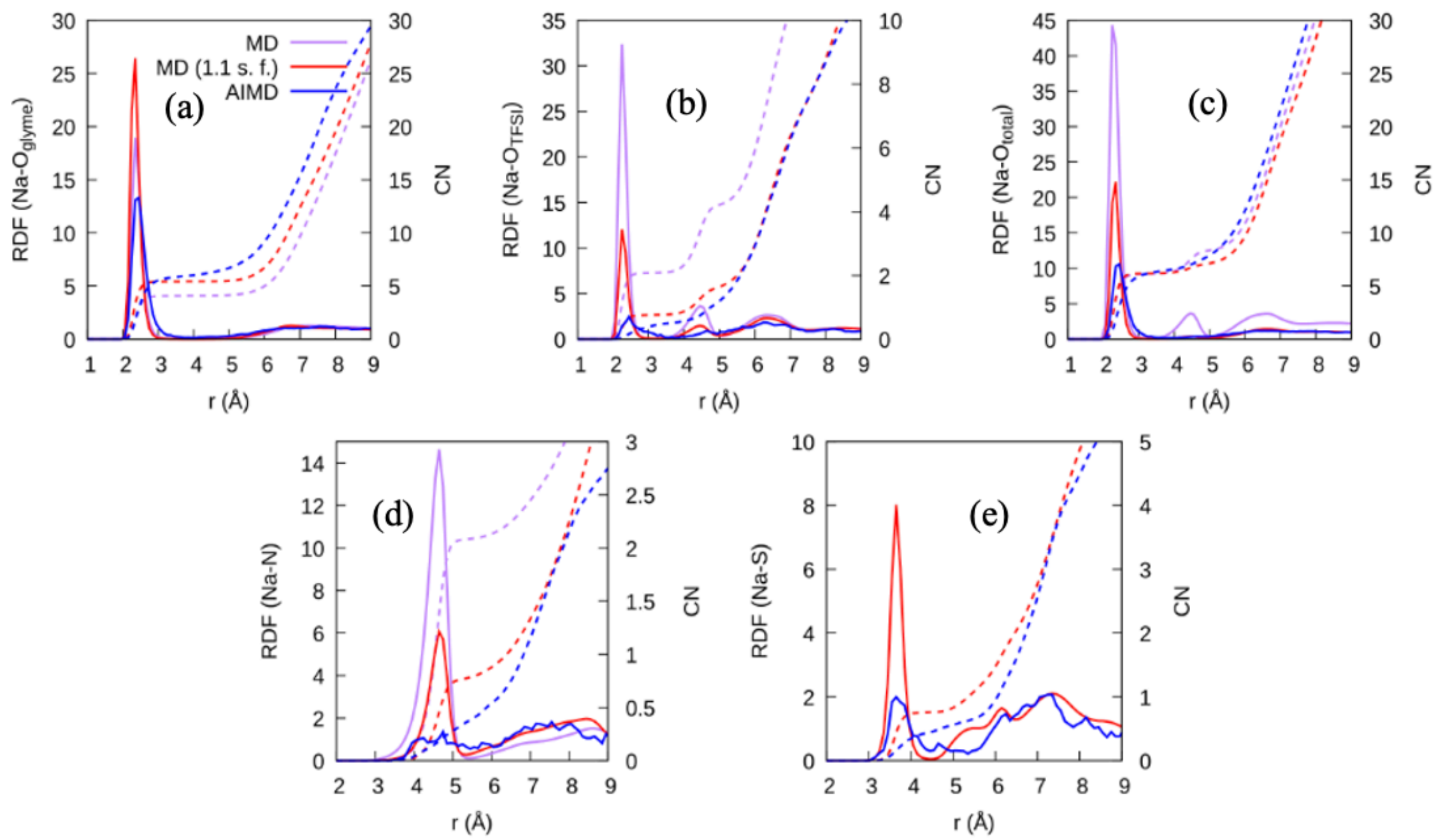

Figure S1. Radial distribution functions (RDFs, solid lines) and cumulative coordination numbers (CNs, dashed lines) for NaTFSI in G1 computed from classical MD with original partial charges (purple), classical MD with a 1.1 charge scaling factor (red), and AIMD (blue) for (a) Na-O$_{glyme}$, (b) Na-O$_{TFSI}$ (c) Na-O$_{total}$, (d) Na-N, and (e) Na-S.

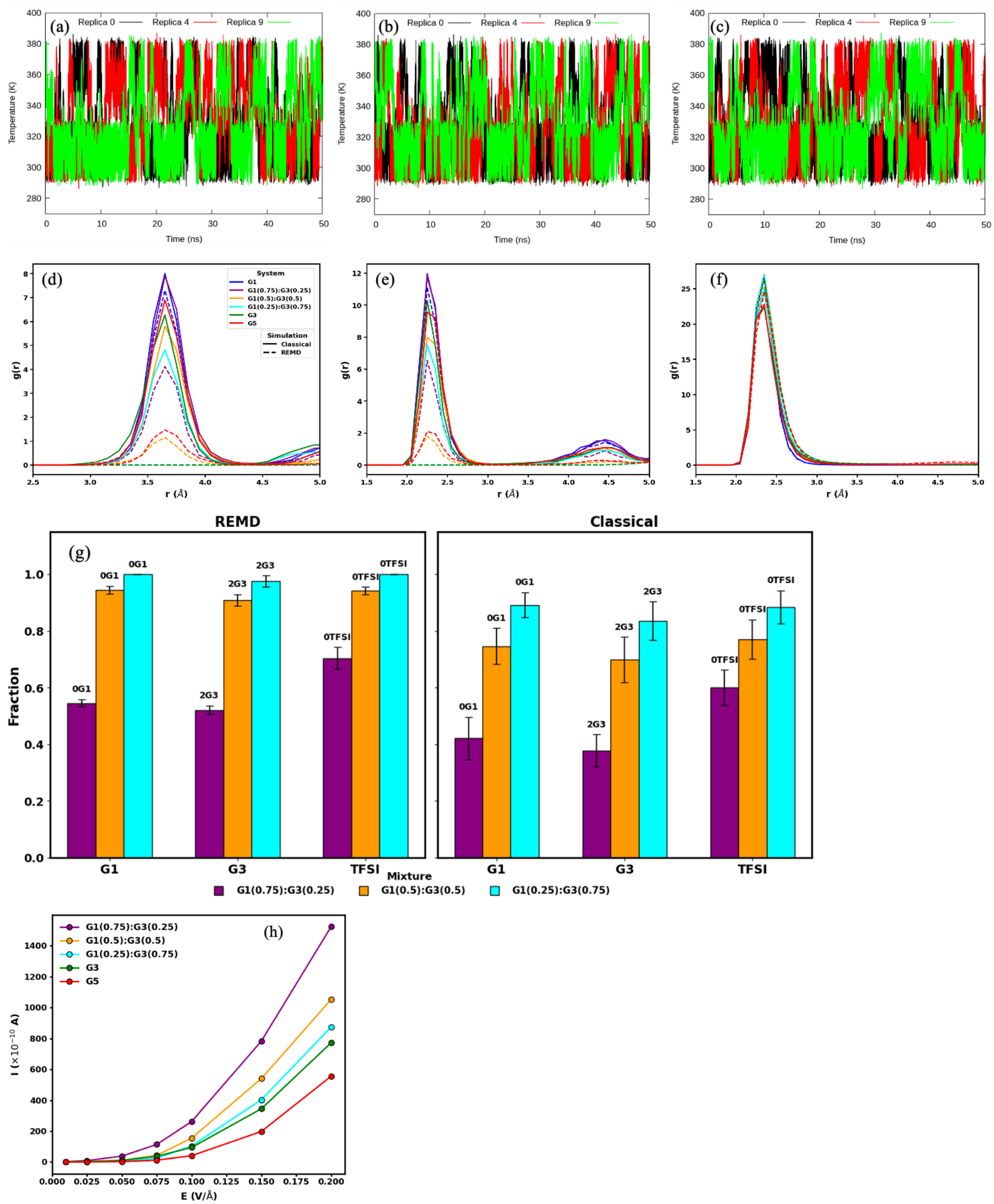

Figure S2. Replica temperature exchange, Na^+ solvation structure, and coordination fraction comparison for the G1:G3 mixed-glyme electrolytes. (a–c) Temperature evolution for three representative replicas:

Replica 0 (300 K, black), Replica 4 (330 K, red), and Replica 9 (370 K, green), during the REMD simulation of (a) G1(0.75):G3(0.25), (b) G1(0.5):G3(0.5), and (c) G1(0.25):G3(0.75). (d–f) Radial distribution functions, g(r), between Na^+ and (d) $TFSI^-$ sulfur, (e) $TFSI^-$ oxygen, and (f) glyme oxygen atoms, for G1, G3, G5, and the three G1:G3 mixtures, comparing classical MD (solid lines) and REMD (dashed lines) at 300 K (g) Dominant Na^+ coordination fractions (0G1, 2G3, 0TFSI) from REMD and classical MD for the three mixtures. (h) Current–electric field relationships for five systems at multiple field strengths (0.01, 0.025, 0.05, 0.075, 0.10, 0.15, and 0.20 V/Å).

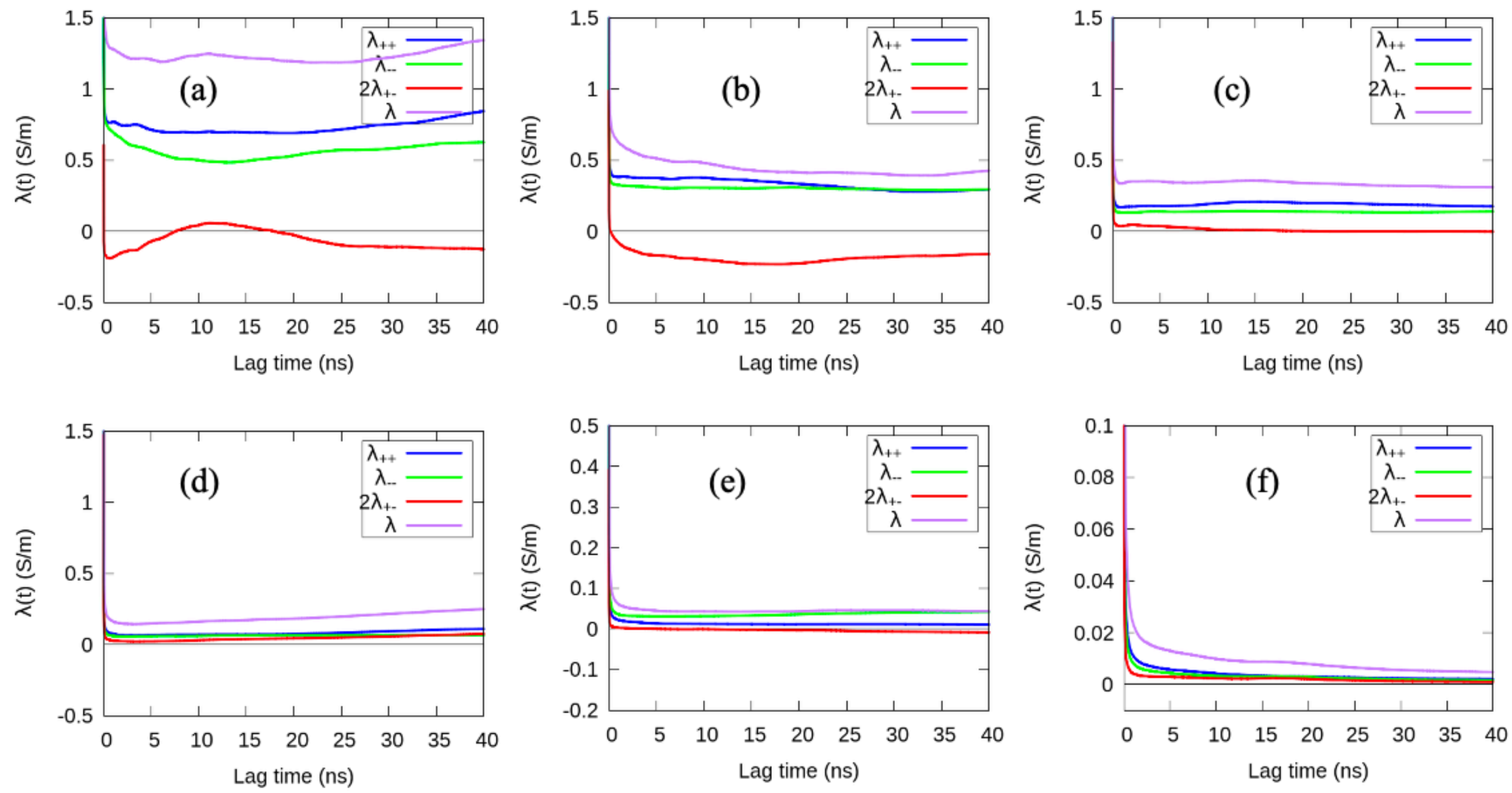

Figure S3. Convergence of the Nerst correlated ionic conductivity components: cation-cation (, blue), anion-anion (, green), cross-term (, red), and total (, purple) as a function of lag time for (a) G1, (b) G1(0.75):G3(0.25), (c) G1(0.5):G3(0.5), (d) G1(0.25):G3(0.75), (e) G3, and (f) G5.

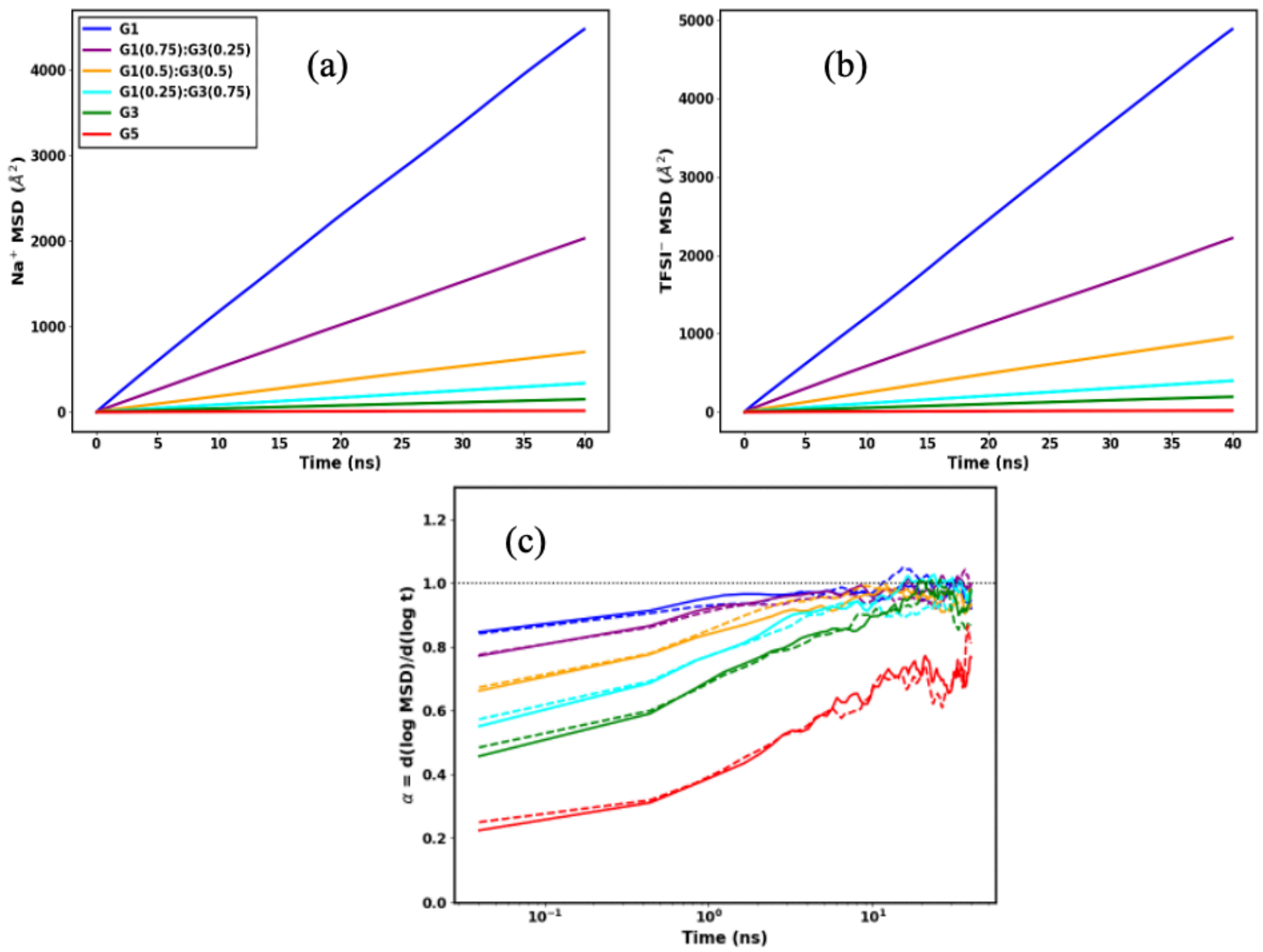

Figure S4. Mean squared displacement (MSD) vs. time for (a) Na^+ and (b) $TFSI^-$ in all six systems. (c) Local diffusion exponent, $\alpha = d(\log MSD)/d(\log t)$, for Na^+ (solid lines) and $TFSI^-$ (dashed lines). $\alpha = 1$ (dotted line) indicates diffusive behavior. All systems reach $\alpha \approx 1$ except G5, which remains sub-diffusive throughout the simulation.
